# Distribution of Archaeal Communities along the Coast of the Gulf of Finland and Their Response to Oil Contamination

**DOI:** 10.3389/fmicb.2018.00015

**Published:** 2018-01-23

**Authors:** Lijuan Yan, Dan Yu, Nan Hui, Eve Naanuri, Signe Viggor, Arslan Gafarov, Sergei L. Sokolov, Ain Heinaru, Martin Romantschuk

**Affiliations:** ^1^Department of Environmental Sciences, University of Helsinki, Lahti, Finland; ^2^Faculty of Science and Technology, Institute of Molecular and Cell Biology, University of Tartu, Tartu, Estonia; ^3^Skryabin Institute of Biochemistry and Physiology of Microorganisms, Russian Academy of Sciences, Pushchino, Russia; ^4^Institute of Environmental Sciences, Kazan Federal University, Kazan, Russia

**Keywords:** archaeal community, oil contamination, coastal water, littoral sediment, Gulf of Finland, co-occurrence network

## Abstract

The Baltic Sea is vulnerable to environmental changes. With the increasing shipping activities, the risk of oil spills remains high. Archaea are widely distributed in many environments. However, the distribution and the response of archaeal communities to oil contamination have rarely been investigated in brackish habitats. Hence, we conducted a survey to investigate the distribution, diversity, composition, and species interactions of indigenous archaeal communities at oil-contaminated sites along the coast of the Gulf of Finland (GoF) using high-throughput sequencing. Surface water and littoral sediment samples were collected at presumably oil-contaminated (oil distribution facilities) and clean sites along the coastline of the GoF in the winter 2015 and the summer 2016. Another three samples of open sea surface water were taken as offshore references. Of Archaea, *Euryarchaeota* dominated in the surface water and the littoral sediment of the coast of the GoF, followed by *Crenarchaeota* (including *Thaumarchaeota, Thermoprotei*, and *Korarchaeota* based on the Greengenes database used). The unclassified sequences accounted for 5.62% of the total archaeal sequences. Our study revealed a strong dependence of the archaeal community composition on environmental variables (e.g., salinity, pH, oil concentration, TOM, electrical conductivity, and total DNA concentration) in both littoral sediment and coastal water in the GoF. The composition of archaeal communities was season and ecosystem dependent. Archaea was highly diverse in the three ecosystems (littoral sediment, coastal water, and open sea water). Littoral sediment harbored the highest diversity of archaea. Oil was often detected in the littoral sediment but rarely detected in water at those presumably contaminated sites. Although the composition of archaeal community in the littoral sediment was sensitive to low-input oil contamination, the unchanged putative functional profiles and increased interconnectivity of the archaeal core species network plausibly revealed resilience and the potential for oil degradation. *Halobacteriaceae* and putative cytochrome P450 pathways were significantly enriched in the oil-contaminated littoral sediment. The archaeal taxa formed highly interconnected and interactive networks, in which *Halobacteriaceae, Thermococcus*, and methanogens were the main components, implying a potential relevant trophic connection between hydrocarbon degradation, methanogenesis, sulfate reduction, and/or fermentative growth.

## Introduction

The classical dictum “Everything is everywhere, but, the environment selects” (Baas-Becking, 1934) is a fundamental theory in studies of prokaryotic communities and their biogeographic distributions (de Wit and Bouvier, 2006; Zhang et al., 2014). It reflects a niche assembly theory, according to which the distribution patterns of microbial species at different scales are highly dependent on ecosystem types explained by physical, chemical, and biological environmental traits (Auguet et al., 2010; Campbell and Kirchman, 2013). However, this niche assembly theory has been challenged by the unified neutral theory (Hubbell, 2001). Microbes that have a small body size and high abundance are subject to dispersal due to wind, waves, and currents in the marine environment (Pommier et al., 2007). Freshwater and marine organisms of different taxonomic groups show varied degrees of spatial autocorrelation patterns, driven by both niche assembly and dispersal processes associated with their biotic traits (e.g., life-history type and dispersal capacity; Astorga et al., 2012; Zhang et al., 2014).

As the third domain of life, the Archaea have been an exciting topic for ecologists. Nevertheless, their uncultivated status, comparatively low abundance, and limited database have restricted studies on their biogeographic distribution and metabolic diversity in various environments. In recent years, a few well-designed 16S rRNA gene-based global and regional studies have successfully characterized the biogeographic distribution patterns and diversity of uncultured archaea in many environments (Auguet et al., 2010; Xie et al., 2014). Many studies have studied the distribution of archaea in brackish ecosystems, however using different methods and scales of resolution. For instance, Levipan et al. (2012) identified two distinct communities of free-living archaea inhabiting continental freshwater and the coastal marine environment along a marine–freshwater transect in central-southern Chile using a fingerprinting technique (PCR-DGGE). Other studies characterized the distribution and roles of ammonia-oxidizing crenarchaeota (AOA) (Francis et al., 2005; Dang et al., 2008) and methanogenic archaea (Wen et al., 2017) in estuaries based on a functional gene approach.

However, the distribution of archaea in the Baltic Sea has not been well addressed. The Baltic Sea is one of the world's largest brackish ecosystems. It is a semi-closed, intercontinental, and non-tidal system dependent on constant freshwater input from continental rivers in the north and saline water input from the North Sea (Dupont et al., 2014). These environmental conditions create a unique habitat, harboring a mixture of marine and freshwater organisms that have adapted to a wide salinity range from 0 to 30.9%0 (Herlemann et al., 2011). The shallow brackish system is very challenging, being heavily exposed to long-term anthropogenic stressors, such as eutrophication, climate warming, and organic pollutants (Kalson et al., 2002; Andersson et al., 2015; Andersen et al., 2017).

Environmental pollution by petroleum hydrocarbons is a major problem worldwide. These oil products contain hundreds or thousands of aliphatic, branched, and aromatic hydrocarbons. Most of them are toxic to living organisms, and in the long term they can cause toxicity, diseases, cell damage, development disorders, and reproduction problems (Jain et al., 2011). Although fewer oil spills have been recorded in recent years, the risk of oil spills in the Baltic Sea is still profoundly present as a result of busy shipping activities (HELCOM, 2010). Collision, grounding, and contact have been the main causes of accidents in the Baltic Sea (HELCOM, 2014). Among the 150 reported accidents in 2013, six cases, i.e., two tankers, two cargo ships, and two other ships, resulted in oil pollution (HELCOM, 2014). However, most oil spills detected in the Baltic Sea are small illegal or accidental spills (HELCOM, 2010) attributable to the intentional emptying of contaminated bilge waters and cleaning of the oil tanks of ships. These small illegal discharges continue to occur, causing significant cumulative effects on the environment (HELCOM, 2010). The heaviest oil contamination in water has been detected in the Gulf of Finland (GoF) and the northern and central Baltic proper (HELCOM, 2010), making these areas valuable for assessing the impact of oil contamination on marine biodiversity. *In situ* biodegradation of hydrocarbons in the Baltic Sea is slow as a result of the low temperature and complex environment conditions (Suni et al., 2007). Understanding the dynamics of archaeal communities and the interactions between the core taxa in response to oil contamination is important for improving bioremediation techniques in oil-contaminated sites.

The impact of oil contamination on archaeal communities in the brackish environment remains poorly understood. So far, the dynamics of archaeal communities in response to oil contamination have mostly been characterized at the microcosm and mesocosm scales (Röling et al., 2004; Orcutt et al., 2010; Jurelevicius et al., 2014; Sanni et al., 2015; Wang et al., 2016). Such experiments under controlled laboratory conditions have produced contradictory implications for the roles of archaea in oil degradation: either the lack of Röling et al. (2004) or presence of Jurelevicius et al. (2014) oil-degradation potential in marine-associated ecosystems. In addition, these studies have provided no indication of the interactions between the co-existing archaeal taxa and their environment. Therefore, we established a survey off the coast of the GoF, with the aims to (i) extract the general temporal–spatial distribution patterns of the indigenous archaeal communities in the surface water and littoral sediment habitats along the coast of the GoF, (ii) evaluate the impact of oil contamination on archaeal communities, (iii) detect the “indicator species” and their putative metabolic pathways for *in situ* oil degradation, (iv) build phylogenetic molecular networks to illustrate the patterns of co-occurrence and relationships of archaeal taxa in oil-contaminated and clean environments, and (v) link the community distribution patterns with environmental variables.

## Materials and methods

### Sampling and wet-lab analysis

**Figure 1:**
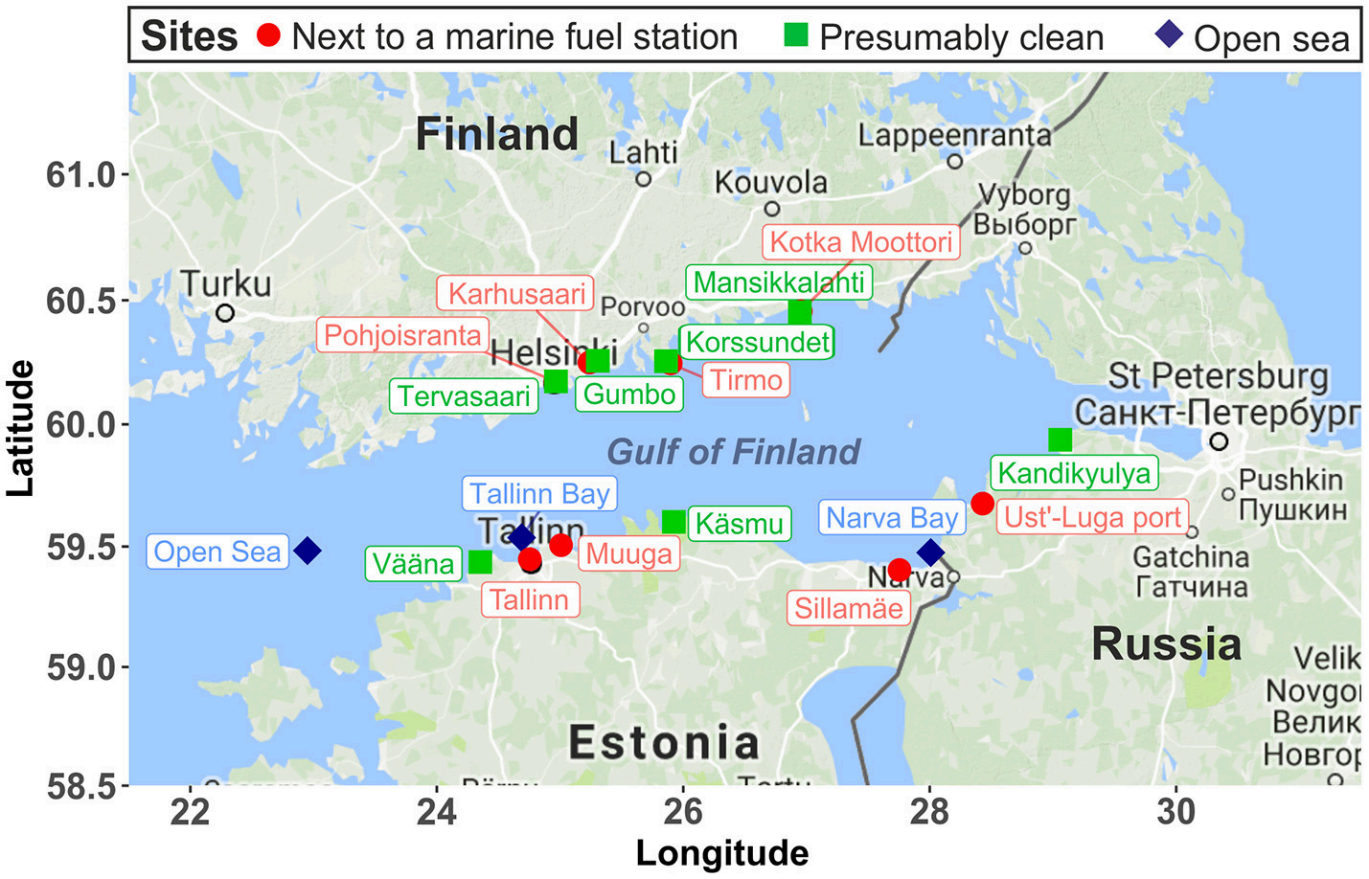
Sampling map (Gulf of Finland). The biochemical properties of these sampling sites were presented in Table 1. The sampling map was obtained from Google Maps (map type: terrain) using the R package *ggmap* (Kahle and Wickham, 2013).

The GoF is a large estuarine basin forming the easternmost arm of the Baltic Sea. It has a low salinity because of the large freshwater input from the continental rivers in the east (Alenius et al., 1998). The GoF is ice-covered from November to April, with an average surface water temperature of 0°C in winter and 15–17°C in summer (Alenius et al., 1998). In our study, we sampled the surface water and littoral sediment (waterlogged, 10–50 cm below the water level) at 14 coastal sites (Figure 1), including 7 presumably contaminated (next to a maritime fuel station) and 7 presumably clean sites, along the coastal area of the GoF in the winter of 2015 and in the summer of 2016. Notably, although Tervasaari was not next to a fuel station, it was probably a contaminated site because of being located in the center of Helsinki, where shipping is intensive. In the summer of 2016, two more sites in Kotka (one presumably contaminated site, Kotka Moottori, and one clean site, Mansikkalahti) were sampled to provide a better coverage of the GoF coastline. In summer, two samples with different soil textures (one with coarse sandy sediment and one with an organic sediment top layer) were taken at Gumbo to evaluate the effect of texture on archaeal communities. Three open sea water samples, Tallinn Bay, Narva Bay, and Open Sea, were taken as reference samples from less human impacted offshore areas. The sampling sites were respectively located ~6, 2, and 37 km from coast. The environmental conditions at these three open sea sites were described in detail by Viggor et al. (2015). Water samples were collected into sterile 1000 mL glass bottles by placing the bottles with the neck near the surface collecting a ca. 5 cm top layer of the surface water. Littoral sediment samples were collected along the water-logged shoreline with three to five dips (subsamples) in sterile 50 mL falcon tubes. In winter, water samples were taken at the ice–water interface. Four replicate samples were taken at each site. Three replicate samples were used for DNA extraction and one for chemical analysis. Water samples were stored at 4°C and analyzed immediately. Sediment samples were thoroughly mixed and stored at −20°C until analysis. Sediment samples were thawed at room temperature and homogenized manually by gently inverting the falcon tubes 20 times prior to chemical analysis and DNA extraction. Chemical analysis of water and sediment samples, including electrical conductivity, pH, salinity, total organic carbon (TOC), total organic matter (TOM), and the concentration of the non-volatile fraction of mineral oil (C10-C40) from petroleum products, was performed with standard procedures by Eurofins Environment Testing Finland Oy, Lahti, Finland. The non-volatile faction of mineral oil (C10-C40) was extracted from sediment and water samples based on the standard procedure ISO 11046:2004, as modified by Jørgensen et al. (2005), and quantified according to ISO 16703:2004.

Prokaryotic cells from 500 mL water were collected on polycarbonate membrane filters (pore size 0.2 μm, diameter 47 mm; Whatman™) prior to DNA extraction. Genomic DNA was extracted from 0.5 g of homogenized sediment samples and from the membrane-filtered water samples using a PowerSoil DNA Isolation kit (MOBIO laboratories, Inc.) according to the manufacturer's instructions. The extracted DNA was quantified using the PicoGreen DNA assay (Invitrogen), as previously described (Yu et al., 2015). The primers used for amplification of the partial 16S rRNA gene (V3-V4 region) were ARC344F (5′-ACGGGGYGCAGCAGGCGCGA-3′) and Arch806R (5′-GGACTACVSGGGTATCTAAT-3′) (Raskin et al., 1994; Takai and Horikoshi, 2000) with the PCR program shown in Supplementary Table S1. All sequences were generated with Illumina's MiSeq platform using paired end reads at the Institute of Biotechnology, University of Helsinki, Finland.

**Table 1:** Summary of the biochemical properties of the GoF sampling sites.

**Ecosystem**	**Site**	**Longitude**	**Latitude**	**Season**	**Country**	**Conductivity (mS/m)**	**Salinity (%0)**	**TOC (mg L^−1^)**	**C10-C40**	**C10-C21**	**C21-C40**	**pH**	**DM**	**TOM**	**DNA (mg g^−1^ or mg L^−1^)**	**No. Seq**	**No. Unclassfied**
									**(mg kg**^**−1**^ **or mg L**^**−1**^**)**								
OS	Narva Bay	28.01	59.48	Winter	EST	625	3.40	–	0	0	0	–	–	–	0.79	608	26
OS	Open Sea	22.95	59.48	Winter	EST	1176	6.70	–	0	0	0	–	–	–	0.51	31	0
OS	Tallinn Bay	24.69	59.54	Winter	EST	1061	6.00	–	0	0	0	–	–	–	0.74	777	30
OS	Narva Bay	28.01	59.48	Summer	EST	730	4.00	6.5	0	0	0	8.00	–	–	2.58	500	6
OS	Open Sea	22.95	59.48	Summer	EST	1100	6.00	7.0	0	0	0	8.10	–	–	1.09	272	20
OS	Tallinn Bay	24.69	59.54	Summer	EST	1000	5.60	5.7	0	0	0	8.23	–	–	3.56	869	58
W	Käsmu	25.92	59.60	Winter	EST	940	5.70	–	0	0	0	7.70	–	–	1.11	121	0
W	Muuga	25.01	59.51	Winter	EST	900	4.30	–	0	0	0	7.80	–	–	1.56	158	0
W	Sillamäe	27.75	59.40	Winter	EST	700	4.00	–	0	0	0	8.50	–	–	1.85	346	0
W	Tallinn	24.75	59.45	Winter	EST	990	5.20	–	0	0	0	8.00	–	–	1.75	93	0
W	Vääna	24.35	59.44	Winter	EST	990	6.00	–	0	0	0	7.60	–	–	0.62	888	0
W	Gumbo	25.31	60.26	Winter	FIN	–	–	–	0	0	0		–	–	0.46	177	11
W	Korssundet	25.86	60.26	Winter	FIN	790	4.37	–	0	0	0	7.60	–	–	0.47	713	2
W	Pohjoisranta	24.96	60.17	Winter	FIN	770	4.25	–	0	0	0	7.80	–	–	5.61	1948	1
W	Tervasaari	24.97	60.17	Winter	FIN	790	4.37	–	0	0	0	7.70	–	–	0.64	356	35
W	Tirmo	25.90	60.25	Winter	FIN	–	–	–	0	0	0		–	–	0.42	117	0
W	Karhusaari	25.24	60.25	Winter	FIN	720	3.96	–	0	0	0	7.80	–	–	4.14	548	90
W	Kandikyulya	29.06	59.94	Winter	RUS	530	2.85	–	0	0	0	7.50	–	–	1.08	93	0
W	Ust'-Luga port	28.43	59.68	Winter	RUS	280	2.85	–	0	0	0	7.10	–	–	1.70	72	0
W	Käsmu	25.92	59.60	Summer	EST	810	4.50	30.0	0	0	0	7.57	–	–	2.71	1524	91
W	Muuga	25.01	59.51	Summer	EST	920	5.10	12.0	0	0	0	7.20	–	–	3.06	1510	19
W	Sillamäe	27.75	59.40	Summer	EST	530	2.90	14.0	0	0	0	7.87	–	–	2.36	657	3
W	Tallinn	24.75	59.45	Summer	EST	980	5.50	6.0	0	0	0	8.18	–	–	4.65	823	260
W	Vääna	24.35	59.44	Summer	EST	1000	5.60	5.9	0	0	0	7.93	–	–	3.91	919	119
W	Gumbo	25.31	60.26	Summer	FIN	900	5.00	6.3	0	0	0	7.80	–	–	1.85	337	35
W	Korssundet	25.86	60.26	Summer	FIN	810	4.50	6.9	0	0	0	7.47	–	–	2.89	857	252
W	Kotka Moottori	26.95	60.46	Summer	FIN	690	3.80	6.9	1	0	1	7.53	–	–	3.11	762	1
W	Mansikkalahti	26.95	60.45	Summer	FIN	650	3.50	7.1	0	0	0	7.60	–	–	2.73	724	0
W	Pohjoisranta	24.96	60.17	Summer	FIN	830	4.60	7.1	0	0	0	7.73	–	–	6.20	646	201
W	Tervasaari	24.97	60.17	Summer	FIN	740	4.10	8.3	0	0	0	7.90	–	–	7.89	1868	228
W	Tirmo	25.90	60.25	Summer	FIN	870	4.80	6.6	0	0	0	7.77	–	–	2.38	424	46
W	Karhusaari	25.24	60.25	Summer	FIN	890	5.00	6.9	0	0	0	7.77	–	–	2.19	197	34
W	Kandikyulya	29.06	59.94	Summer	RUS	420	2.30	11.0	0	0	0	7.33	–	–	6.91	579	0
W	Ust'-Luga port	28.43	59.68	Summer	RUS	510	2.70	13.0	0	0	0	7.47	–	–	2.87	242	0
S	Käsmu	25.92	59.60	Winter	EST	89	–	–	220	0	130	6.60	0.20	0.48	14.23	839	0
S	Muuga	25.01	59.51	Winter	EST	38	–	–	0	0	0	7.50	0.82	0.01	0.22	86	1
S	Sillamäe	27.75	59.40	Winter	EST	36	–	–	0	0	0	7.40	0.79	0.02	0.28	97	0
S	Tallinn	24.75	59.45	Winter	EST	64	–	–	24	0	19	8.90	0.82	0.02	0.16	94	0
S	Vääna	24.35	59.44	Winter	EST	37	–	–	0	0	0	8.70	0.81	0.01	0.57	35	0
S	Gumbo	25.31	60.26	Winter	FIN	–	–	–	94	24	71	–	0.57	0.09	16.39	252	3
S	Korssundet	25.86	60.26	Winter	FIN	–	–	–	0	0	0	–	0.81	0.02	0.67	333	4
S	Pohjoisranta	24.96	60.17	Winter	FIN	–	–	–	700	370	330	–	0.83	0.06	4.13	320	0
S	Tervasaari	24.97	60.17	Winter	FIN	–	–	–	210	89	120	–	0.85	0.02	0.64	151	0
S	Tirmo	25.90	60.25	Winter	FIN	–	–	–	0	0	0	–	0.85	0.01	1.04	196	0
S	Karhusaari	25.24	60.25	Winter	FIN	–	–	–	92	24	68	–	0.84	0.02	0.52	224	1
S	Kandikyulya	29.06	59.94	Winter	RUS	27	–	–	0	0	0	7.40	0.83	0.02	0.05	115	0
S	Ust'-Luga port	28.43	59.68	Winter	RUS	29	–	–	11	0	0	6.70	0.71	0.06	1.66	71	0
S	Käsmu	25.92	59.60	Summer	EST	54	–	–	0	0	0	6.90	0.81	0.01	0.85	580	1
S	Muuga	25.01	59.51	Summer	EST	53	–	–	0	0	0	9.40	0.82	0.01	0.76	506	5
S	Sillamäe	27.75	59.40	Summer	EST	15	–	–	0	0	0	7.80	0.80	0.01	2.73	244	0
S	Tallinn	24.75	59.45	Summer	EST	59	–	–	21	0	15	9.30	0.85	0.01	0.38	211	0
S	Vääna	24.35	59.44	Summer	EST	62	–	–	0	0	0	9.70	0.80	0.01	1.56	203	1
S	Gumbo	25.31	60.26	Summer	FIN	39	–	–	0	0	0	8.30	0.90	0.04	0.48	367	1
S	Gumbo organic	25.31	60.26	Summer	FIN	130	–	–	67	0	47	6.50	0.16	0.38	45.65	390	1
S	Korssundet	25.86	60.26	Summer	FIN	64	–	–	0	0	0	7.30	0.77	0.01	2.13	654	1
S	Kotka Moottori	26.95	60.46	Summer	FIN	48	–	–	48	30	18	6.50	0.70	0.02	1.31	345	10
S	Mansikkalahti	26.95	60.45	Summer	FIN	42	–	–	0	0	0	6.60	0.76	0.01	0.39	348	2
S	Pohjoisranta	24.96	60.17	Summer	FIN	76	–	–	400	140	260	7.50	0.76	0.04	4.69	568	37
S	Tervasaari	24.97	60.17	Summer	FIN	51	–	–	19	0	14	8.30	0.80	0.01	1.03	527	10
S	Tirmo	25.90	60.25	Summer	FIN	70	–	–	0	0	0	9.20	0.79	0.01	0.51	340	11
S	Karhusaari	25.24	60.25	Summer	FIN	71	–	–	21	0	11	7.40	0.79	0.01	0.32	333	20
S	Kandikyulya	29.06	59.94	Summer	RUS	57	–	–	0	0	0	6.10	0.77	0.02	3.23	354	3
S	Ust'-Luga port	28.43	59.68	Summer	RUS	32	–	–	0	0	0	8.30	0.83	0.00	0.62	385	1

*S, littoral sediment; W, coastal water; OS, open sea water; TOC, total organic carbon in water; Conductivity, electrical conductivity; Salinity, water salinity; C10-C40, non-volatile faction of mineral oil (C10-C40) concentration; C10-C21, non-volatile faction of mineral oil (C10-C21) concentration; C21-C40, non-volatile faction of mineral oil (C21-C40) concentration; DM, dry matter content of sediment; TOM, total organic matter of sediment; DNA, total genomic DNA concentration extracted and measured as a proxy of total microbial biomass in water and sediment samples; No. Seq, number of sequences retrieved per site; No. Unclassified, number of unclassified sequences per site*.

### Bioinformatics analyses

The sequence data of partial bacterial 16S rRNA gene amplicons were analyzed using the mothur pipeline (Version 1.35.1) (Schloss et al., 2009) according to MiSeq SOP (Kozich et al., 2013). For quality control, the sequences that contained ambiguous (N) bases and homopolymers longer than 8 nucleotides were screened out. The remaining sequences were pre-clustered to allow for up to one difference per 100 bp to reduce potential sequencing errors before the identification of the chimeric sequences using the UCHIME algorithm. After removing the chimeric sequences, the unique sequences were classified with the mothur-formatted Greengenes taxonomy reference database (gg_13_8_99, http://www.mothur.org/w/images/6/68/Gg_13_8_99.taxonomy.tgz), using the default bootstrapping algorithm (cutoff value: 80%). *Thaumarchaeota* and *Korarchaeota* are newly proposed archaeal phyla (Elkins et al., 2008; Stieglmeier et al., 2014). In this study, these two new phyla were assigned as classes within the phylum *Crenarchaeota* according to the Greengenes taxonomy reference database used. As the taxonomic classification only matters on the description of the taxonomic composition of certain archaea and had no impact on the following statistical interpretation, we decided to keep the classification assigned based on the Greengenes database used. Non-archaeal sequences were removed. The remaining 29,924 archaeal sequences were then clustered into 727 operational taxonomic units (OTUs) using the OptiClust clustering algorithm at 97% similarity. Half of the OTUs (374) were singletons. The unidentified archaeal reads only accounted for 5.62% of the total quality-filtered sequences. The singletons were therefore kept for further analysis, assuming that their occurrence was a natural pattern with the implication that the corresponding species were inherently rare in the studied environment, instead of PCR biases or sequencing errors. The sequencing data were deposited in the NCBI Sequence Read Archive (SRA) database under accession no. SRP113711.

### Statistical analysis

The samples in which oil was empirically detected were re-categorized as oil-contaminated samples (Detected). Clean samples were categorized as below the detection level (BDL).

#### Linking to environmental variables

Bray-Curtis distance-based redundancy analysis (db-RDA) was used to investigate the relationships between the observed archaeal communities and measured environmental variables using the R package *vegan* (Oksanen et al., 2007). As TOC was only measured in summer samples, separate db-RDA analysis was performed using all summer data. The *P*-values were calculated based on 9,999 permutations. Variables that formed significant linear relationships with archaeal communities were plotted on db-RDA plots. The relationships between the measured environmental variables were tested using Pearson correlation. The relationships between the relative abundance of archaeal taxa (classes) and the measured environmental variables were determined using Spearman rank correlation with *P*-values adjusted according to Benjamini and Hochberg (1995).

#### Spatial autocorrelation

To strengthen the reliability of our assumptions on the impact of oil contamination on archaeal communities in the GoF, we performed spatial autocorrelation analysis in the R package *ape* (Paradis et al., 2016). The geographic distance matrix was computed based on “Vincenty” ellipsoid great circle distances using the R package *geosphere* (Hijmans et al., 2016). Mantel's statistic was used to test for the correlation between the geographic distance matrix and the biological distance (weighted UniFrac) matrix using 9,999 permutations, with the null hypothesis that the similarity of the archaeal community structure of the same ecosystem between two sites was not affected by their spatial distance. Moran's I was used to test for spatial autocorrelation in the relative abundance of the major archaeal classes (relative abundance >0.5%).

#### Beta diversity

The relative abundance of each OTU was calculated by dividing the read count of this OTU by the total read count of the corresponding sample, prior to beta diversity analysis. Principle coordinate analysis (PCoA) was performed on the relative abundance data based on the weighted UniFrac distance using the R package *phyloseq* (McMurdie and Holmes, 2013). PERMANOVA was used to assess whether the *a priori* groups of ecosystem, season, and oil detection and their interaction resulted in a different archaeal community composition with the default 999 permutations. Betadisper was used to test whether the dispersions of observations between *a priori* groups were equal with 999 permutations. PERMANOVA and Betadisper were performed based on the Bray-Curtis distance using the R package *vegan* (Oksanen et al., 2007). Distance-based discriminant analysis (db-DA) was performed to confirm and visualize the significant differences in the community composition between the *a priori* groups based on Bray-Curtis distance using the R package *BiodiversityR* (Kindt and Kindt, 2017) with 9,999 permutations.

#### Predicted functional community and biomarker discovery

Phylogenetic investigation of communities by reconstruction of unobserved states (PICRUSt, Langille et al., 2013) was used to predict the functional composition of the observed archaeal communities. NSTI values were calculated (mean = 0.09) to evaluate the sufficiency of the reference genomes in covering the metagenome to generate accurate prediction, prior to a PICRUSt run. Discriminant analysis was performed to test the effect of oil contamination on the putative functional composition of the observed archaeal communities based on the Euclidean distance with 9,999 permutations using the R package *BiodiversityR* (Kindt and Kindt, 2017). The linear discriminant analysis effect size (LEfSe, Segata et al., 2011) was used to determine the differentially abundant taxa at all taxonomic levels and the putative KEGG pathways (i) between oil-contaminated (Detected) and clean sites (BDL), irrespective of the season (Class: oil detection; Subclass: season) and (ii) between winter and summer (Class: season) in water and sediment.

#### Network analysis

The random matrix theory (RMT)-based approach (Zhou et al., 2011; Deng et al., 2012) was followed using the MENA pipeline (http://ieg2.ou.edu/MENA) to construct phylogenetic molecular ecological networks (pMENs) of oil contaminated (Detected) and clean (BDL) core archaeal taxa in the littoral sediment samples. As the input data, we used the relative abundance of the observed archaeal OTUs of the *a priori* groups multiplied by a constant of 100,000 to make the minimal input value over 1. The similarity matrix was then constructed based on data that were not logarithmically transformed using Spearman's rho. The constructed pMENs were visualized using Cytoscape software v.3.2.1.

## Results

### Taxonomic composition of archaeal communities in the GoF

**Figure 2:**
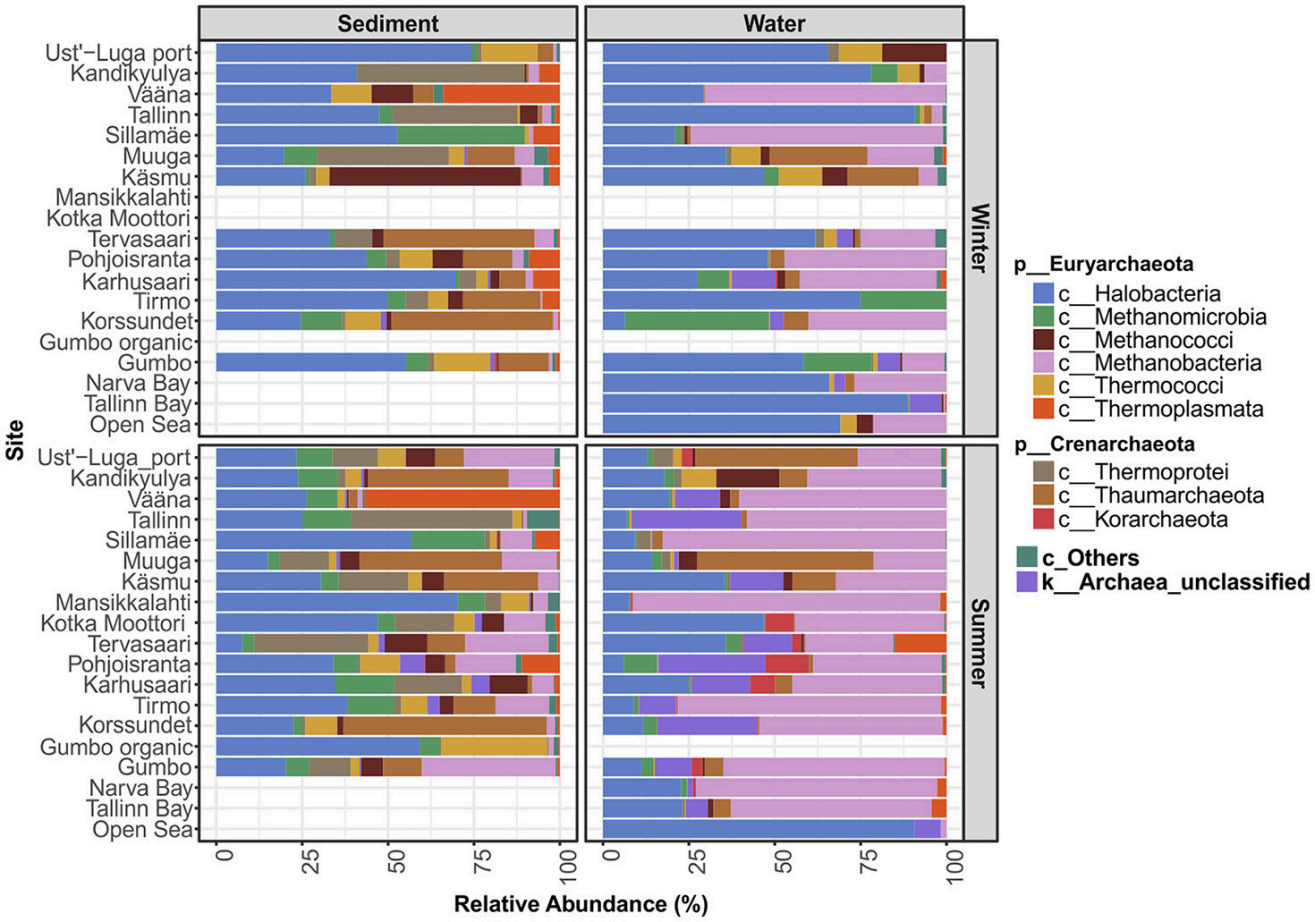
Archaeal community composition of different sites at the class level. “c_Others” represents a sum of rare classes (including *Archaeoglobi, Micrarchaea*, pOWA133, pISA9, AAG, MHVG, THSCG, unclassified *Euryarchaeota* and *Crenarchaota*), which showed a mean relative abundance value <0.5% individually. The new archaeal phyla *Thaumarchaeota* and *Korarchaeota* are shown as classes within the phylum *Crenarchaeota* in this study according to the Greengenes taxonomy reference database used.

The number of archaeal sequences retrieved and the number of unclassified archaeal sequences at each sampling site are presented in Table 1. Out of the total of 29,924 archaeal sequences sampled, 727 OTUs were assigned (462 in the littoral sediment, 378 in the coastal water, and 77 in the open sea water). The library sizes ranged from 31 to 1,948 per site (Table 1). At the phylum level, *Euryarchaeota* comprised the majority (78.58%) of the total sequences, followed by *Crenarchaeota* (15.76% including *Thermoprotei, Korarchaeota*, and *Thaumarchaeota* based on the Greengenes database used), with the remaining 5.62% of sequences being unclassified archaea. At the class level (Figure 2), *Halobacteria* was ubiquitous and predominant in all samples, with an average relative abundance of 58.25% in the open sea water, 31.15% in the littoral sediment, and 37.65% in the coastal water. *Methanobacteria* was the second largest class, and was detected with a significantly higher abundance in water (40.50% in the coastal water and 31.44% in the open sea water) than in the littoral sediment (8.03%). *Thaumarchaeota* formed the third largest population in our samples, representing 13.93% of the total archaeal sequences detected in the littoral sediment, 7.68% in the coastal water, and 1.41% in the open sea water. Unclassified archaeal sequences accounted for an average abundance of 7.86% in the coastal water, 1.01% in the littoral sediment, and 4.53% in the open sea water. As shown in Figure 2, the littoral sediment harbored highest diversity of archaea among the three ecosystems (littoral sediment, coastal water, and open sea water). The rare *Crenarchaeota* classes (i.e., with relative abundance lower than 0.5%) of pISA9, MHVG, THSCG, and pOWA133 were only present in the littoral sediment, whereas AAG only appeared in the coastal water (data not shown).

There were clearly site-specific variations in the taxonomic composition of archaeal communities (Figure 2). For instance, in the sediment samples taken at Vääna, *Thermoplasmata* showed high relative abundance in both winter and summer.

### Environmental selection of archaeal populations and spatial autocorrelation

#### Environmental variables at the sampling sites of the GoF

Environmental variables at different sites were measured to explain the site-specific patterns of archaeal taxonomic composition (Table 1). Environmental variables exhibited significant latitudinal and longitudinal variability in both water (Supplementary Table S2) and sediment (Supplementary Table S3). In water, changes in pH values were significant as a function of both longitude and latitude. Salinity, which was highly correlated with electrical conductivity, was negatively correlated with longitude in water. Salinity in the GoF was low, ranging from 2.30%0 in the east to 6.70%0 in the west (Table 1). The Russian sites that are close to the large continental rivers showed the lowest salinity. The open sea samples showed the highest salinity, except the samples taken in the Narva Bay close to the Narva River. In the littoral sediment, electrical conductivity, the oil concentration, pH, and TOM were all negatively correlated with longitude. Electrical conductivity and the oil concentration were positively correlated with latitude, whereas pH was negatively correlated with latitude in the littoral sediment. The total organic matter (TOM) was highest in Käsmu, in which the littoral sediment was mainly composed of dead algae in winter. In water, the DNA concentration was on average highest in Pohjoisranta (Helsinki, Finland) and lowest in the open sea site; in sediment, the DNA concentration was positively correlated with TOM (Supplementary Table S3). Changes in the microbial DNA concentration in water samples were not pronounced in association with either longitude or latitude.

**Figure 3:**
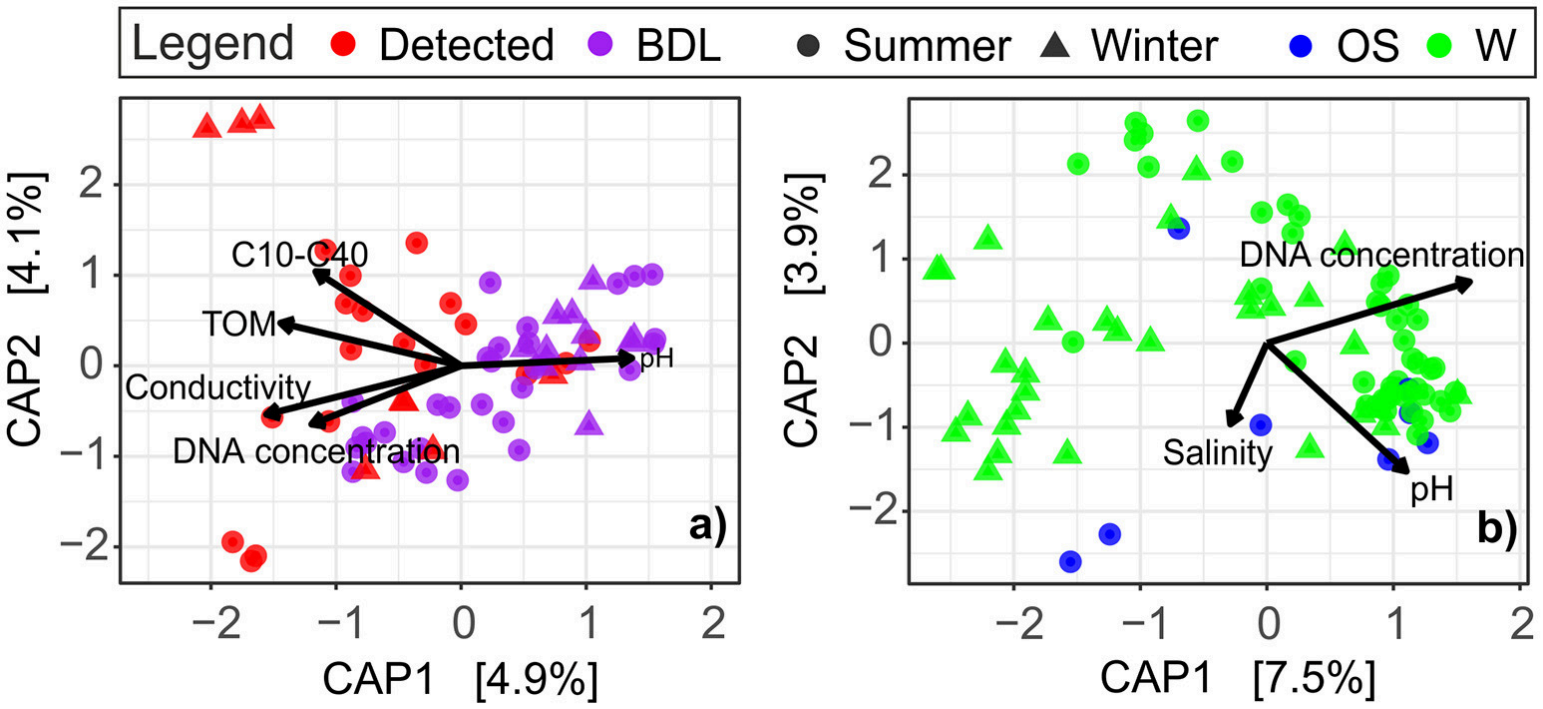
Visualization of the relationships between the observed archaeal communities and the measured environmental variables in **(a)** littoral sediment and **(b)** surface water at the GoF, based on distance-based redundancy analysis (db-RDA, scaling = 2). The marginal test result of the effect of each individual environmental variable on the observed archaeal communities is presented in Supplementary Table S4. W, coastal water; OS, open sea water; BDL, oil concentration below detection level (clean samples); Detected, oil detected (oil-contaminated samples).

Distance-based redundancy analysis revealed a strong dependence of the archaeal community composition on environmental variables in both sediment and water (Figure 3 and Supplementary Table S4). In db-RDA plots, the ordination axes were constrained to linear combinations of the measured environmental variables. In the littoral sediment, the oil concentration, TOM, electrical conductivity, and DNA concentration were significantly positively correlated with the oil-contaminated samples. pH was the only environmental variable that showed a significant negative association with oil-impacted communities. These significant variables accounted for 17.81% of the total variation in archaeal communities in the littoral sediment (Figure 3a). In the surface water, the variations in archaeal community composition were not pronounced in relation to oil contamination, but were in relation to electrical conductivity/salinity, pH, and the DNA concentration, with a total of 21.28% of the variance explained by these environmental variables (Figure 3b). TOC had a positive correlation with water samples taken close to river estuaries in the summer, particularly those Russian samples taken close to the Luga River (Supplementary Figure S1).

#### Spatial autocorrelation patterns of archaeal communities

**Figure 4:**
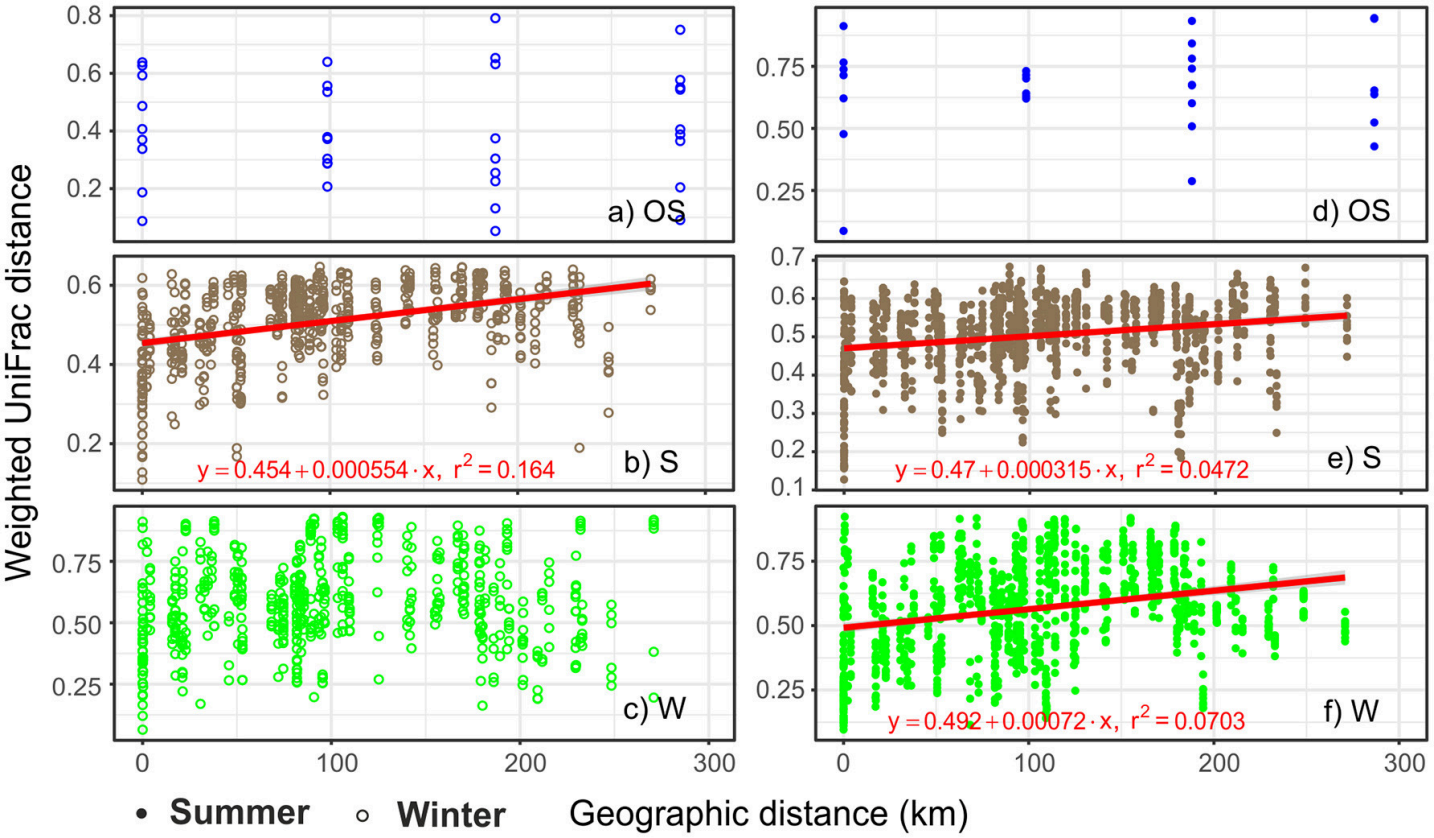
Ecosystem and season dependent distance-decay patterns of archaeal communities in the GoF. Mantel's statistic was used test the pairwise correlation between the geographic distance matrix and the biological distance matrix (weighted UniFrac). Mantel's *P*-values were calculated based on 9,999 random permutations. Mantel's *P*-values are 0.43 **(a)**, 1e-04 **(b)**, 0.21 **(c)**, 0.24 **(d)**, 2e-04 **(e)**, 8e-04 **(f)**, respectively. The observed mantel correlation *r*-values are 0.02 **(a)**, 0.40 **(b)**, 0.07 **(c)**, 0.13 **(d)**, 0.22 **(e)**, and 0.27 **(f)**, respectively. A linear regression model was fitted if the mantel correlation was significant (*P* < 0.05). The slope of the linear regression was calculated to reveal the rate of distance-decay. S, littoral sediment; W, coastal water; OS, open sea water.

Mantel tests of the correlation between pairwise biological distances and geographic distances showed significant spatial autocorrelation patterns of archaeal communities in the littoral sediment and the coastal water, but yielded no significant results in the open sea water (Figure 4). The littoral sediment communities displayed a strong distance-decay pattern with high mantel correlation *r*-values, irrespective of the season; nevertheless, the spatial autocorrelation in archaeal communities in the coastal water was season dependent, in terms of only being significant in summer. Based on Moran's I test of the relative abundance of individual major classes, only *Methanomicrobia* had a positive spatial autocorrelation in both seasons (Supplementary Table S5).

In order to link the spatial autocorrelation patterns with the measured environmental variables, correlation analysis between environmental factors and archaeal classes was performed. In the open sea water and littoral sediment, none of the archaeal classes displayed a correlation between their relative abundance and measured environmental variables. In the coastal water, there were more correlations between the relative abundance of archaeal classes and the measured environmental gradients (Table 2). For instance, *Methanobacteria* was negatively correlated with TOC, whereas *Thermoprotei* was positively correlated with TOC; *Thermoprotei* was negatively correlated with electrical conductivity and salinity, while *Korarchaeota* was positively correlated with the oil concentration. However, none of the classes were correlated with pH, irrespective of the ecosystem.

**Table 2:** The correlations between observed relative abundance of archaeal taxa (classes) and environmental variables.

**Class**	**Ecosystem**	**TOC**	**Conductivity**	**Salinity**	**C10-C40**
c__Korarchaeota	Coastal water	–	–	–	0.38^**^
c__Methanobacteria	Coastal water	−0.43^*^	–	–	–
c__Thermoprotei	Coastal water	0.61^***^	−0.35^*^	−0.37^*^	–
k__Archaea_unclassified	Coastal water	−0.48^*^	0.35^*^	0.39^**^	–

*** BH adjusted P < 0.001;

** BH adjusted P < 0.01;

* *BH adjusted P < 0.05. – BH adjusted P > 0.05. Spearman's correlation rho values were given only when the BH adjusted P-values were lower than 0.05. TOC, total organic carbon in water (unit: mg L^−1^); Conductivity, electrical conductivity (unit: mS/m); Salinity, water salinity (unit: %0); C10-C40, non-volatile faction of mineral oil (C10-C40) concentration (unit: mg L^−1^)*.

### Season and ecosystem-dependent patterns of archaeal communities

#### Structural difference in archaeal communities between seasons

**Figure 5:**
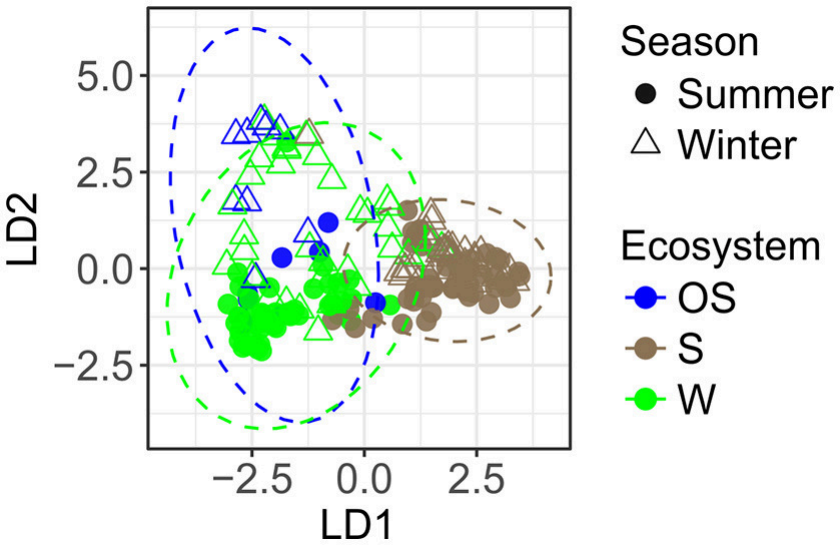
Variation of archaeal communities among different ecosystems and seasons, based on Bray-Curtis distance-based discriminant analysis (db-DA, *a priori* group: EcoSeason, perm. *P* < 0.001). The 95% confidence ellipses of the *a priori* groups were displayed in dotted circles assuming a multivariate normal distribution. S, littoral sediment; W, coastal water; OS, open sea water.

The PCoA ordination displayed a broad pattern of variation between archaeal communities (Supplementary Figure S2). The pairwise test of PERMANOVA revealed significant differences in archaeal community structures between the coastal water, the littoral sediment, and the open sea water (*P* < 0.001, Supplementary Table S6). Distance-based discriminant analysis characterized significantly different patterns of archaeal communities between ecosystems on the first axis and season on the second axis (*P* < 0.001, Figure 5 and Supplementary Table S6).

#### Season-specific archaeal taxa

**Figure 6:**
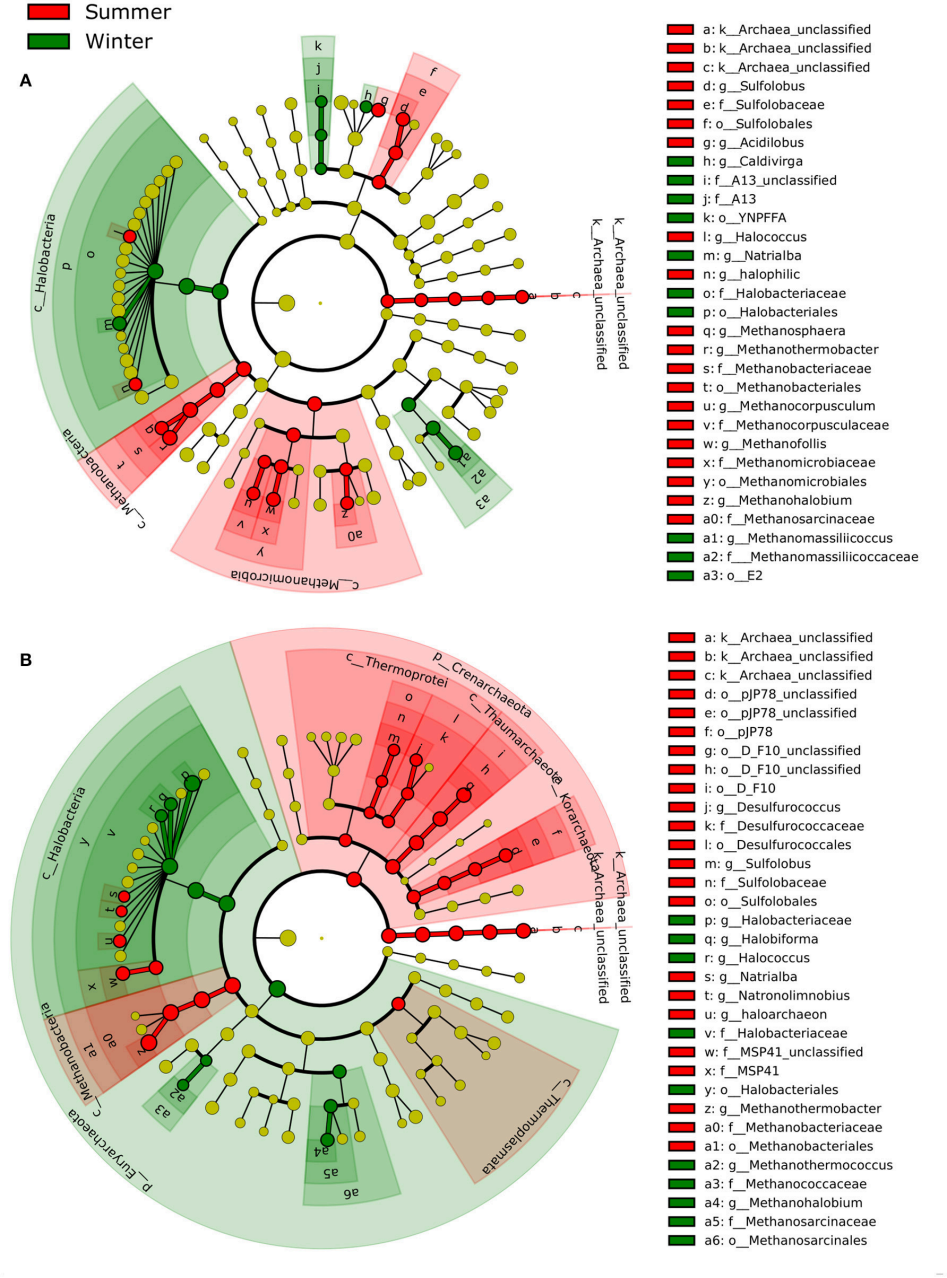
Seasonality of archaeal communities in **(A)** littoral sediment and **(B)** surface water (at both coastal and open sea sites) based on LEfSe analysis (*P* < 0.05, LDA effect size >2). The archaeal taxa with increased relative abundance in summer were shaded in red and those with increased relative abundance in winter in green. The new archaeal phyla *Thaumarchaeota* and *Korarchaeota* are indicated as classes within the phylum *Crenarchaeota* in this study according to the Greengenes taxonomy reference database used.

LEfSe identified several archaeal taxa that displayed differing preferences (i.e., increase or decrease in relative abundance in the community) between summer and winter (Figure 6). In the littoral sediment, two classes, *Methanomicrobia* (e.g., *Methanohalobium, Methanofollis*, and *Methanocorpusculum*) and *Methanobacteria* (e.g., *Methanothermobacter* and *Methanosphaera*), and two genera of *Sulfolobus* and *Acidilobus* favored summer, whereas the predominant class, *Halobacteria*, the family A13 and the genus *Methanomassilliicoccus* showed a preference for winter. In water, *Crenarchaeota* (e.g., *Thermoprotei, Thaumarchaeota, Korarchaeota*) generally preferred summer, whereas *Euryarchaeota* was more winter-favoring, with some exceptions. For example, two *Euryarchaeota* classes, *Thermoplasmata* and *Methanobacteria*, had a preference for summer.

### Effect of oil on archaeal communities

#### Oil detection

The results of chemical analysis of the samples are presented in Table 1. Among the studied sites, total petroleum hydrocarbons (C10-C40) were detected at low concentrations in the sediment and water at a few sites. In the littoral sediment, oil was constantly detected at Pohjoisranta, Tervasaari, Gumbo, Karhusaari, and Tallinn in both winter and summer seasons, although the concentrations were higher in winter than in summer. Pohjoisranta (in the Helsinki area) was the most contaminated, with 700 mg kg^−1^ of oil detected in winter and 400 mg kg^−1^ in summer. The neighboring site, Tervasaari, was also slightly contaminated, with 210 mg kg^−1^ detected in winter and 19 mg kg^−1^ in summer. Oil was more often detected in sediment than in water. In water, only 0.08 mg L^−1^ of oil was detected at Karhusaari and 0.25 mg L^−1^ at Ust'-Luga Port in winter, and 0.16 mg L^−1^ at Pohjoisranta and 1.40 mg L^−1^ in Kotka Moottori in summer.

#### The effect of oil on the structure of archaeal communities and the oil-responding taxa

**Figure 7:**
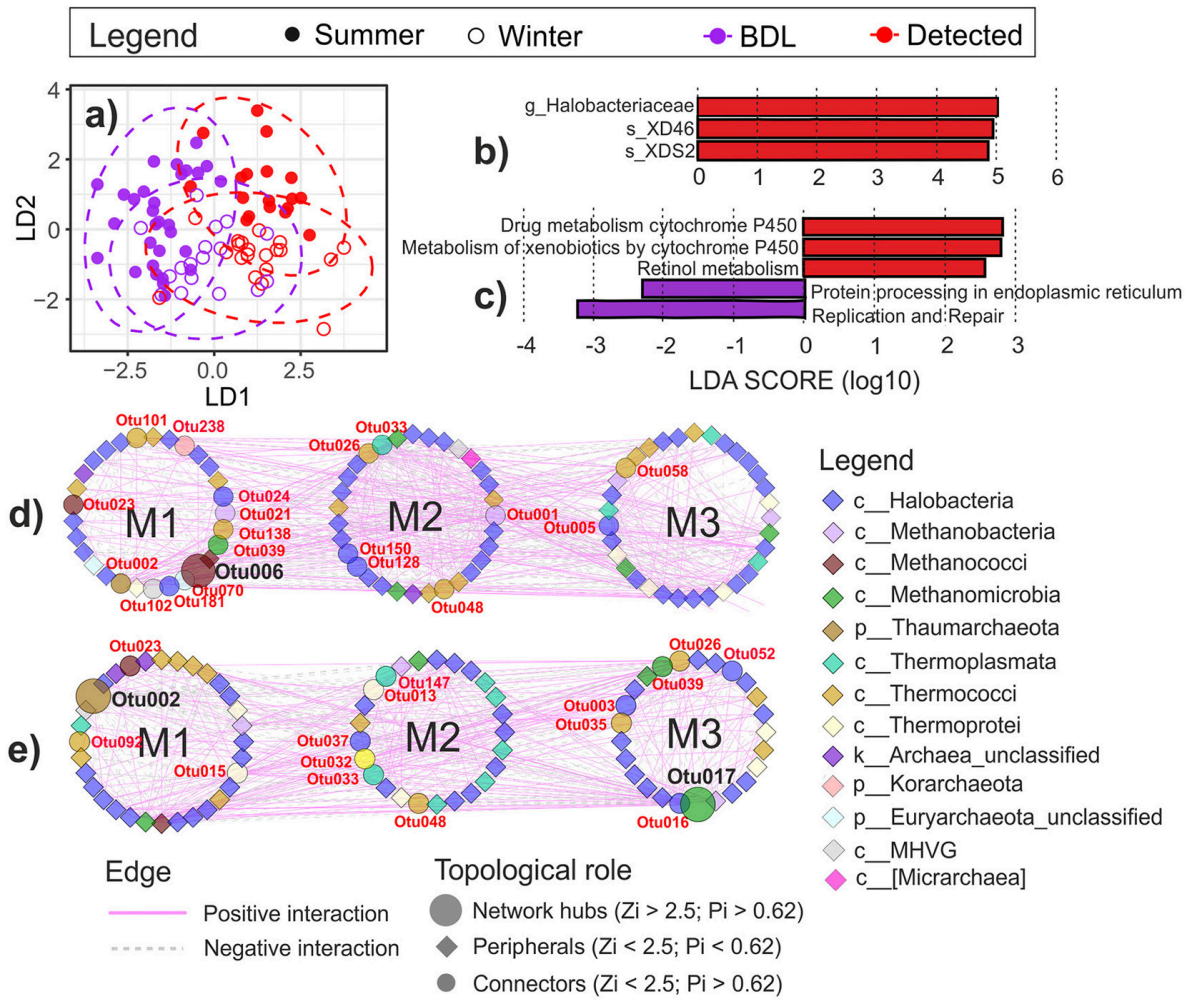
Changes in archaeal communities in response to oil contamination in the littoral sediment at the coast of the GoF. **(a)** Visualization of the difference of archaeal communities between oil-contaminated and clean littoral sediment samples in different seasons (*a priori* group: OilSeason) using Bray-Curtis distance-based discrimination analysis. The 95% confidence ellipses of the *a priori* groups were displayed in dotted circles assuming a multivariate normal distribution. Oil-responding biomarkers of **(b)** archaeal taxa and **(c)** predicted KEGG pathways using LEfSe analysis (*P* < 0.05, LDA effect size > 2). The oil-favoring biomarkers were displayed in red and the oil-depressed ones in purple. Phylogenetic molecular ecological networks (pMENs) of archaeal OTU interactions in the oil-contaminated **(d)** and clean **(e)** littoral sediment. The networks were visualized using “module number” as the group attributes layout. Modules were displayed with “M” followed with the number in each pMEN. The detailed taxonomy of each node is presented in Supplementary Table S7. S, littoral sediment; W, coastal water; OS, open sea water; BDL, oil concentration below detection level (clean samples); Detected, oil detected (oil-contaminated samples). The new archaeal phyla *Thaumarchaeota* and *Korarchaeota* are indicated as classes within the phylum *Crenarchaeota* in this study according to the Greengenes taxonomy reference database used.

No impact of oil on the archaeal community composition was observed in the coastal water, but the impact was significant in the littoral sediment in both seasons (Figure 7a). Therefore, LEfSe analysis was performed only on the littoral sediment data. In the littoral sediment, *Halobacteriaceae* (in particular, species XD46 and XDS2) was significantly more abundant in the presence of oil (Figure 7b).

#### The effect of oil on the predicted functional composition of archaeal communities and the oil-responding putative functional pathways in the littoral sediment

PICRUSt analysis produced a high relative abundance of putative pathways such as amino acid metabolism, energy metabolism, translation, and membrane transport (Supplementary Figure S3). Less than 20% of the pathways were unclassified. Oil had no impact on the putative metabolic functional composition in the archaeal communities in water or in the littoral sediment (db-DA perm. *P* > 0.05, based on the Euclidean distance). Nevertheless, LEfSe detected two oil-enhanced cytochrome P450 pathways (drug metabolism—cytochrome P450 and metabolism of xenobiotics by cytochrome P450) and Retinol metabolism at the molecular level in the sediment (Figure 7c).

#### The effect of oil on the co-occurrence network in the littoral sediment

Phylogenetic molecular ecological network analysis was used to illustrate the co-occurrence of core OTUs and their associations. As no oil effect was detected in water, only pMENs in oil-contaminated (Figure 7d) and clean littoral sediment (Figure 7e) were constructed, irrespective of the season. Taxonomy of the core OTUs (nodes) that constructed the oil-contaminated and the clean pMENs is shown in Supplementary Table S7. The network properties of the two empirical pMENs and randomized networks are presented in Table 3. Both pMENs exhibited scale-free characteristics, with the fitted R square of the power law being 0.810 in oil-contaminated and 0.805 in clean littoral sediment. Both networks had small-world behaviors, as indicated by the average geodesic distances (path lengths), which were close to the logarithms of the total number of nodes. Both pMENs were modular, as their modularity values were significantly higher than those of their corresponding randomized networks. Three modules were detected in both pMENs. Oil contamination induced distinctly different network structures in archaeal communities. The oil-contaminated pMEN was more complex and had closer-linked nodes (OTUs) than the clean pMEN, with a significantly higher clustering coefficient, higher modularity, and lower geodesic distance (path length). The predominant *Halobacteria* (including *Halobacteriaceae* and MSP41) comprised 51.61 and 50%, methanogens (including *Methanobacteriaceae, Methanocaldococcaceae, Methanocorpusculaceae, Methanomicrobiaceae, Methanosarcinaceae*, and *Methermicoccacea*) 12.90 and 12.20%, and *Thermococci* 16.12 and 15.85% of the total nodes in contaminated and clean pMENs, respectively. Only 50 (29%) nodes were shared by the two pMENs. At the class level, one *Micrarchaea*, one *Korarchaeota* (order pJP78), and two unclassified *Euryarchaeota* nodes were only present in the contaminated pMEN. Regarding the topological roles, 20% of the nodes were connectors in the oil-contaminated pMEN and 18% in the clean pMEN. There was one network hub (OTU006: *Methanocaldococcus*) in the contaminated pMEN and two (OTU002: *Thaumarchaeota* order D-F10 and OTU017: *Methanofollis liminatans*) in the clean pMEN. Neither pMENs had module hubs. The rest of the nodes were peripherals (78% in the contaminated and 79% in the clean pMEN).

**Table 3:** Topological properties of the empirical oil-contaminated and clean pMENs and their corresponding random pMENs in the littoral sediment of the GoF.

**Oil**	**Empirical networks**									**Random networks (Mean** ±***SD*****)**		
	**No. of original OTUs^a^**	**Similarity threshold (S_t_)^b^**	**Network size^c^**	***R*^2^ of Power law**	***pp/np*^d^**	**Avg connectivity**	**Avg geodesic distance**	**Avg clustering coefficient**	**Modularity (No. of modules)**	**Avg geodesic distance**	**Avg clustering coefficient**	**Avg modularity**
Contaminated	315	0.310	93	0.810	316/304	13.333	2.001^e^	0.462^e^	0.235 (3)^e^	2.031 ± 0.013	0.381 ± 0.015	0.168 ± 0.008
Clean	277	0.310	82	0.805	234/225	11.195	2.004^e^	0.451^e^	0.233 (3)^e^	2.017 ± 0.014	0.430 ± 0.018	0.181 ± 0.008

a *The number of archaeal OTUs that were originally used for network construction using RMT-based approach*.

b *If two OTUs have a correlation value larger than the similarity threshold, this correlation is highly significant according to Fisher transformation test*.

c *The number of OTUs (e.g., nodes) in a network*.

d *The number of positive correlations or edges (pp) and negative correlations (np) detected in a pMEN. In this correlation-based network, a positive (or negative) co-occurrence relationship unveils the same (or opposite) trend of changes in relative abundance between a pair of OTUs of interest*.

e *Significant difference (P < 0.001) of the indices between contaminated and clean pMENs. The means and standard deviations (SD) of individual indices were computed based on the 100 times of randomly rewiring the network connections. The standard deviations were also used for the Student t-test of the significance of these network properties between the oil-contaminated and clean pMENs. Avg, average*.

## Discussion

### Ecosystem dependency of archaeal communities

Both water and sediment harbored diverse archaeal populations in the GoF, although half of the archaeal OTUs observed were singletons with a site-specific occurrence. The GoF archaeal communities were dominated by *Euryarchaeota* in both water and sediment, in accordance with a survey along the coast of the South China Sea (Xie et al., 2014). *Halobacteria*, methanogens (e.g., *Methanobacteria, Methanomicrobia*, and *Methanococci*), and *Thaumarchaeota* were present with a high relative abundance, indicating the adaptation of these taxa to the brackish environment. The archaeal community structure significantly differed between ecosystems, suggesting varied habitat preferences among archaeal species.

The archaeal community was most diverse in the littoral sediment, as the littoral sediment is a highly heterogeneous and complex habitat. Cross-contamination of species through sampling was observed at the interface of water and sediment, i.e., sediment shared 130 archaeal OTUs with the ambient coastal water in the present study. As the coastal water and the littoral sediment are highly connected ecosystems, it is inevitable that they seed each other's diversity (Helmus et al., 2007; Shade, 2017).

As expected, the archaeal community structure of open sea water significantly differed from that of coastal water. It highly agrees with the finding that the archaeal assemblages differ between the coastal and the central Arctic Ocean (Galand et al., 2006). In estuarine and coastal sea, it is inevitable to find bacteria and archaea that are of freshwater (river) or soil origins (e.g., Dang et al., 2008, 2010a,b, 2013). Coastal water is a more heterogeneous environment than open sea water as a result of a more complex mixture of organic substrates, particles and nutrients bioavailable for microbial heterotrophy (Galand et al., 2006). The input of both the particle-associated and free-living microbes from the connected rivers and soils likely enriches microbial composition in the coastal sea water, contributing to a different microbial community structure (Dang and Lovell, 2015). In turn, the river-borne and soil-borne microbes may serve as bio-tracers of the riverine and/or wastewater discharges in the coastal sea water (e.g., Dang et al., 2008, 2010b).

### Linking archaeal community changes to environmental variables and seasonality

Archaeal communities were significantly affected by environmental variables that changed along certain geographic gradients in the GoF. In the coastal water, the DNA concentration (as a proxy of microbial biomass), pH, salinity, and electrical conductivity were significant drivers of the changes in archaeal communities. These environmental variables were responsible for the observed seasonal patterns, represented by the separation of summer and winter archaeal communities in the db-RDA ordination. The db-RDA analysis also revealed that the seasonal pattern in water was associated with salinity and total DNA concentration. The coastal water of GoF exhibited lower salinity and higher total DNA concentration in summer than in winter. Salinity alone explained a small but significant proportion (3.36%) of the variation in archaeal communities in both seasons. The total DNA concentration, used as a proxy of microbial biomass, alone explained a higher proportion (5.11%) of the variation than salinity (3.36%) in archaeal communities. Salinity has also previously been reported as a major driver of the global and regional distribution of uncultured archaea (Auguet et al., 2010; Xie et al., 2014). The narrow salinity range across the GoF and the good adaption of archaeal species in such a brackish environment might have limited the detection of the effect of salinity on archaeal communities to some extent.

Correlations with environmental factors reveal the conditions favoring or disfavoring specific groups of organisms (Steele et al., 2011), although correlation analysis alone is not enough to tell the cause-effect relationships. Among all major classes tested, the crenarchaeotal class *Thermoprotei* was negatively correlated with salinity in water, likely suggesting its freshwater preference, in accordance with Xie et al. (2014). In summer, TOC played an important role in shaping archaeal communities in water. Riverine discharge is the main source of TOC in the water of the GoF (Kulinski and Pempkowiak, 2011). As the Russian sites were located close to the estuary of the Luga River, the positive correlation between the Russian sites and TOC could indirectly confirm the riverine origin of the TOC. It could also explain why the freshwater taxon *Thermoprotei* was positively correlated with TOC in the coastal water. Therefore, the large riverine input of freshwater-borne *Thermoprotei*, low salinity and high TOC might together explain the observed seasonality of archaeal composition detected in water, i.e. higher relative abundance of *Crenarchaeota* and the lower relative abundance of *Euryarchaeota* (mainly *Halobacteria*) in summer with regard to those in winter. Other survey on the archaeal community in the southern North Sea reveals that most *Crenarchaeota* are nitrifiers and that nutrient (e.g., ammonium, nitrate, nitrite, and phosphate) concentrations, phytoplankton abundance and composition are the determinants of the abundance of *Crenarchaeota* (Herfort et al., 2007). The unmeasured environmental variables, such as water temperature and light, are likely the primary drivers of seasonality in the marine microbiome (Ward et al., 2017).

In the littoral sediment, TOM, the oil concentration (C10-C40), electrical conductivity, the DNA concentration, and pH were important in determining the archaeal community composition, which is in line with a microbiome study conducted in coastal sediment habitats contaminated by chronic PAHs (Jeanbille et al., 2016). *Korarchaeota* showed a positive correlation with oil concentration. *Korarchaeota* is collectively uncultured and poorly characterized, with little available information on its ecological functions (Reysenbach et al., 2000). Therefore, its apparent preference for oil deserves further investigation. The relative abundance of other archaeal classes was not correlated with oil concentration, indicating their resistance to oil contamination. Although pH (ranging from 6.1 to 9.7) was a significant driver of archaeal communities in both sediment and water, no classes displayed significant correlations with it, plausibly indicating the compositional complexity of archaeal species, with varied preferences for pH in each class. The seasonal patterns in the littoral sediment might not be explained by the measured environmental variables, as the separation of winter and summer observations was not clear in the db-RDA plot. The seasonal patterns of the brackish archaeal communities in the GoF is of great ecological implications, therefore deserving further multi-year observation.

### Spatial autocorrelation patterns of archaeal community

Spatial autocorrelation may violate assumptions tested by standard statistical methods, if the observations are not independent of one another (O'Brien et al., 2016). The mantel test revealed a significant distance-decay relationship in archaeal communities of the GoF. Sediment-borne communities displayed a strong distance-decay relationship, as reflected by the higher Mantel r values. This is consistent with previous research demonstrating that the microbes are more spatially restricted or dispersal is limited in more heterogeneous environments such as soils than in well-mixed ecosystems such as the aquatic environment (Bell, 2010; Gibbons et al., 2013; O'Brien et al., 2016). The coastal water-borne archaeal communities showed significant spatial autocorrelation as a distance-decay pattern in summer, but not in winter. Dispersal on marine sediments and patterns of microbial biogeography are driven by the connectivity of local water masses to ocean circulation (Müller et al., 2014). Large terrestrial and riverine inputs of nutrients and freshwater from the easternmost part of the GoF (Kulinski and Pempkowiak, 2011) might have considerably changed the physicochemical properties of the marine water and sediment locally and strengthened the dispersal of archaeal species, resulting in the distance-decay patterns of archaeal communities in the coastal area, especially in summer. No spatial autocorrelation was detected in the open sea water, which is probably attributable to stochasticity and the low sampling effort compared with the coastal sediment and water. Among the major classes, only *Methanomicrobia* showed significant spatial autocorrelation in the coastal water, irrespective of the season; however, the relative abundance of *Methanomicrobia* was not correlated with latitude, longitude, or with any of the measured environmental variables. Hence, the non-random distribution of *Methanomicrobia* might either be accidental due to the small number of samples used for the analysis, as it was not spatially autocorrelated in the open sea samples, or be associated with unmeasured environmental variables, processes and mechanisms in the coastal water.

### The impact of oil contamination on archaeal communities

Characterizing archaeal communities in the presence of oil contamination in the coastal area is an import step toward understanding the biodegradation potential of oil hydrocarbons in the unique brackish environment. Oil contamination had different effects on the composition of archaeal communities in the coastal water and sediment. In the coastal water, as the oil concentrations were low and oil was detected at less than half of the sites, it was not surprising that oil had no significant impact on archaeal communities. In the littoral sediment, although detected in low concentrations, oil contamination significantly altered the composition of archaeal communities. This is in contrast to the results of the microcosm and mesocosm studies, in which oil contamination was reported to have no impact on the composition or diversity of marine archaeal communities (Orcutt et al., 2010; Sanni et al., 2015). Notably, oil contamination was detected more frequently in the urban areas, such as Helsinki, indicating a mainly anthropogenic origin of oil contamination at these sites. Surprisingly, oil or oil-like substances were occasionally detected in organic-rich sediment samples at presumably clean sites such as Gumbo and Käsmu. Naturally-occurring hydrocarbons, e.g., from algae and plant waxes (Laflamme and Hites, 1979; Volkman et al., 1992; Yamane et al., 2013), were probably the main source of oil detected at these sites.

### Oil-favoring taxa and putative metabolic pathways in the littoral sediment

*Halobacteriaceae*, a well-known family of halophilic archaea, were ubiquitous and predominant in both water and sediment in the GoF. They can degrade a wide range of hydrocarbons in hypersaline environments through mechanisms such as the production of hydrocarbon-degrading enzymes (Erdogmuş et al., 2013) and surface-active agents (Post and Al-Harjan, 1988). Hence, the higher abundance of *Halobacteriaceae*, particularly XD46 and XDS2, at the oil-contaminated than at the clean sites was plausibly associated with *in situ* oil degradation in the littoral sediment. Under low oxygen conditions, *Halobacteria* are also able to utilize red pigment-mediated ATP synthesis to initiate hydrocarbon degradation (Al-Mailem et al., 2012). Owing to phototrophic growth, *Halobacteria* has considerable potential for *in situ* anaerobic hydrocarbon degradation in brackish environments continuously contaminated by low-input oil.

This study putatively defined the gene contents and the metabolic potential of the archaeal communities using PICRUSt analysis, offering insights into how marine archaeal communities respond to oil contamination in the GoF. Oil induced no impact on the predicted functional composition, plausibly suggesting a functional resilience of the observed community in response to low-input oil contamination in the sediment. Cytochromes P450 are a superfamily of enzymes that comprise many oxidase systems in *Bacteria, Archaea*, and *Eukarya*. Cytochrome P450 monooxygenases are highly involved in the aerobic degradation of hydrocarbons in species of bacteria, fungi and fish in various environments (Lee and Anderson, 2005; van Beilen et al., 2006; Syed et al., 2013; Fuentes et al., 2014). Although the function of this enzyme in archaea is not yet known, the significant enrichment of putative cytochrome P450 pathways in an oil-impacted community probably suggests the potential of the P450-encoding archaeal groups in the community to biodegrade oil. Here, we only present results from the putative analysis of functional traits of the observed archaeal communities, with possible horizontal gene transfer of oil-degrading elements between microbes not being considered.

### Oil-induced changes in archaeal pMENs of the littoral sediment

Microbes live in association (e.g., mutualism, antagonism, and competition) with each other in a particular habitat. The co-occurrence and interactions of individual taxa within the community are not assumed to be random, but the taxa are presumed to be dependent on each other, as well as on their surrounding environment. Therefore, phylogenetic molecular ecological networks (pMENs) based on random matrix theory (RMT) were used to illustrate the co-occurrence patterns and the interactions of the core sediment archaeal populations in the presence and absence of oil contamination. The reliability and sensitivity of an RMT-based approach in soil and seawater have been previously characterized (Zhou et al., 2011; Deng et al., 2012; Zhang et al., 2014; Weiss et al., 2016; Wang et al., 2017). In the littoral sediment, the observed non-random co-occurrence patterns might be a result of species or genes that share identical or complementary functions and require similar environmental conditions and ecological niches for habitation (Zhou et al., 2011). In the littoral sediment, both oil-contaminated and clean pMENs exhibited common features observed in other complex ecological networks, such as scale-free, small world, and modular properties (Olesen et al., 2007; Deng et al., 2012).

Oil contamination significantly increased the complexity and modularity of the archaeal pMEN. The contaminated pMEN exhibited a shorter average path (geodesic distance), a higher connectivity, and a higher average clustering coefficient than the clean pMEN, indicating that it was a more efficient system and faster in the transmission of information, energy, and resources. Many complex natural communities exhibit non-random modular networks, in which the tightly connected species are organized together as modules (Olesen et al., 2007). These modules are assumed to be of great structural and functional importance to system stability and ecological resilience (Olesen et al., 2007; Zhou et al., 2010). The increased modularity indicated a denser connection between the core taxa within each module, but a sparse connection between modules in the presence of oil contamination. In addition to the identical module number and similar ratio value between positive and negative interactions, the substantial changes in network topology overall implied a resistant archaeal network in the presence of low-input oil contamination in the littoral sediment of the GoF. However, this result contradicts Jeanbille et al. (2016), who reported a high vulnerability of Bacteria-Archaea-Eukarya networks to PAH contamination in the coastal sediment of the Mediterranean Sea and French Atlantic Ocean, as reflected by oil-depressed network properties.

On closer examination of the taxonomic composition of the nodes, *Halobacteriaceae, Thermococcus*, and methanogens were the main components in both networks. *Halobacteria* includes species such as *Halobacterium, Halococcus*, and *Halorubrum*, which are able to degrade both aliphatic and aromatic hydrocarbons (Al-Mailem et al., 2010; Erdogmuş et al., 2013), but also have the metabolic capacity for assimilatory nitrate reduction in hypersaline environments (Alcántara-Hernández et al., 2009). The genus *Thermococcus* is comprised of sulfur-reducing hyperthermophilic archaea that have a good stress response system (Fukui et al., 2005). Studies have demonstrated that anaerobic hydrocarbon degradation is associated with sulfate reduction, fermentative growth, and/or methanogenesis in oil-contaminated marine environments, without requiring exogenous electron acceptors (Orcutt et al., 2010; Kotlar et al., 2011; Lu et al., 2012; Campeão et al., 2017). Therefore, the observed co-occurrence patterns of these three taxa with high abundance in the pMENs probably implies potential relevant syntrophic connections between *in situ* hydrocarbon degradation, sulfur reduction, nitrate reduction, and methane production in the littoral sediment.

To characterize the topological roles, the nodes are categorized into four classes with regard to their within-module degree (*Zi*) and among-module degree (*Pi*) values: peripherals, connectors, module hubs, and network hubs (Olesen et al., 2007; Deng et al., 2012). Peripherals represent specialists, whereas module hubs and connectors are generalists, and network hubs are super-generalists (Olesen et al., 2007). Hubs and connectors deserve more attention in the structure of a network, as they are probably responsible for the exchange of resources, e.g., information, energy, and nutrients, within and among the modules. The extinction of these core species would result in the breakdown of the related modules, as well as the relevant ecological functions (Olesen et al., 2007). More than one-fifth of the OTUs identified were structurally important as connectors and network hubs in both pMENs, revealing a high connectivity of the nodes within and among the modules and potentially high resilience of the networks in response to stress. The shift of network hubs from *M. liminatans* (OTU017) and *Thaumarchaeota* (OTU002, D-F10 order) to *Methanocaldococcus* (OTU006) in response to oil contamination might reflect a functional change in the core archaeal community of the littoral sediment. These three OTUs co-occurred in both pMENs, but played different roles. *M. liminatans* and *Methanocaldococcus* are both hydrogenotrophic methanogens (Zellner et al., 1999; Johnson and Mukhopadhyay, 2005). *Methanocaldococcus* is able to produce methane coupled with sulfite reduction (Johnson and Mukhopadhyay, 2005). It is phylogenetically close to *Methanococcus*, which dominates in deep-sea oil reservoirs (Kotlar et al., 2011; Lu et al., 2012), plausibly explaining why it was more structurally important in the oil-contaminated pMEN. *Thaumarchaeota*, known to comprise ammonia-oxidizing archaea (Pester et al., 2011), was probably involved in nitrogen transmission within and between modules in the clean pMEN. Interestingly, although quite abundant in the community, only one *Thaumarchaeota* OTU (OTU002) formed tight connections with other core OTUs in the pMENs. While not being a network hub, this OTU was still of great structural importance as a connector between modules in the oil-contaminated pMEN.

In addition to oil contamination, many other biotic and abiotic factors, processes and mechanisms likely contributed to the detected co-occurrence patterns of the microorganisms in the highly heterogeneous littoral sediment. For example, total bacteria, virus counts, temperature, salinity, nutrient, and redox status were significant factors to affect the co-occurrence network of the marine bacterioplankton (Fuhrman and Steele, 2008). In addition, certain bacteria and archaea are found prone to surface-associated living (e.g., on living animals, algal surfaces, particles, and aggregates of different types and sizes); in this way these organisms may take advantage of enhanced organism interactions and metabolic collaboration to facilitate social behaviors in microbial communities (Dang and Lovell, 2015). Therefore, the co-occurrence of certain archaea might be attributable to their surface association behaviors in the littoral sediment in our study.

## Conclusions

The present study sheds light on the distribution of archaeal communities in oil-contaminated brackish environments. The composition of archaeal communities in the coastal area of the GoF were ecosystem and season dependent. The biogeographic distribution of archaeal taxa was regulated by both environmental traits and dispersal ability. On closer examination, archaeal communities exhibited a distance-decay relationship associated with the environmental gradient in the GoF. Oil was continuously detected at low concentrations in the littoral sediment in both seasons. Oil contamination significantly altered the composition and network properties of archaeal communities in the littoral sediment, probably revealing important implications for ecosystem resilience in response to continuous low-input oil contamination. Our results revealed an oil-degradation potential of indigenous archaeal communities in the littoral sediment of the GoF, which has not previously been characterized.

We anticipate that future studies using whole metagenome sequencing will lead to a much more comprehensive understanding of microbial ecology in the brackish environment of the Baltic Sea than at present. Emerging patterns in the biogeographical distribution of microbial populations will help to guide future pollution control and biodiversity management in the coastal area of the Baltic Sea. Long-term monitoring of contaminated sites is needed to characterize the degradation potential of the observed community for risk control. In the future, archaea and other organisms, particularly bacteria and fungi, should be integrated into a complex oil–microbe interaction network to illustrate the bioremediation patterns along the coast of the Baltic Sea.

## Author contributions

MR, AH, DY, EN, SV, AG, and SS: Designed the study; MR, AH, LY, DY, EN, SV, AG, and SS: Participated in the sampling; LY, NH, and DY: Conducted the microbiological and chemical analysis of the samples; LY: Analyzed the data and prepared the manuscript. All the authors critically revised the manuscript.

### Conflict of interest statement

The authors declare that the research was conducted in the absence of any commercial or financial relationships that could be construed as a potential conflict of interest.
