# Supplementary material for: Distribution of Archaeal Communities along the Coast of the Gulf of Finland and Their Response to Oil Contamination
Source: Front Microbiol. 2018 Jan 23;9:15. doi: 10.3389/fmicb.2018.00015 (PMC5787342; doi:10.3389/fmicb.2018.00015)
Supplement: Supplementary file 1 [file DataSheet1.DOCX]

Supplementary Material

**Distribution of archaeal communities along the coast of the Gulf of Finland and their response to oil contamination**

**Lijuan Yan^*^, Dan Yu, Nan Hui^*^, Eve Naanuri, Signe Viggor, Arslan Gafarov, Sergei L. Sokolov, Ain Heinaru, Martin Romantschuk**

*Correspondence: Lijuan Yan, [lijuan.yan@helsinki.fi](mailto:lijuan.yan@helsinki.fi); Nan Hui, [nan.hui@helsinki.fi](mailto:nan.hui@helsinki.fi)

**1. Supplementary Tables**

**Supplementary Table S1.** PCR program used to amplify 16S rRNA gene for MiSeq sequencing

| Reagents | 1 reaction (µL) |  | PCR program | | |
| --- | --- | --- | --- | --- | --- |
| 5x HF Buffer | 5 |  | 1. | 98 °C | 30 s |
| dNTPs (10 mM) | 0.5 |  | 2. | 98 °C | 10 s |
| ARC344F (10 µM) | 1.25 |  | 3. | 58 °C | 30 s |
| Arch806R (10 µM) | 1.25 |  | 4. | 72 °C | 15 s |
| Phusion HS II polym. 2 U/µL | 0.25 |  | 5. | go to 2. 29 times | |
| MQ | 14.75 |  | 6. | 72 °C | 10 m |
| Template DNA | 2 |  | 7. | 4 °C | ∞ |
| Total | 25 |  |  |  |  |

**Supplementary Table S2.** Results of Pearson correlation and significance level between the biogeochemical variables of the sampling sites in the surface water (including both coastal water and open sea water)

| Variables | Electrical conductivity | Salinity | TOC | C10-C40 | pH | Longitude | Latitude |
| --- | --- | --- | --- | --- | --- | --- | --- |
| Salinity | 1.00*** |  |  |  |  |  |  |
| TOC | -0.28* | -0.28* |  |  |  |  |  |
| C10-C40 | -0.17 | -0.14 | -0.11 |  |  |  |  |
| pH | 0.44*** | 0.50*** | -0.36** | -0.19 |  |  |  |
| Longitude | -0.88*** | -0.92*** | 0.24 | 0.15 | -0.31** |  |  |
| Latitude | -0.23* | -0.20 | -0.27* | 0.31** | -0.32** | 0.05 |  |
| DNA concentration | -0.16 | -0.19 | -0.08 | 0.07 | 0.03 | 0.06 | 0.19 |

Significance level: ***** P < 0.001, **** P < 0.01, *** P < 0.05.

**Supplementary Table S3.** Results of Pearson correlation between the biogeochemical variables of the sampling sites in the littoral sediment

| Variables | Electrical conductivity | C10-C40 | pH | TOM | Longitude | Latitude |
| --- | --- | --- | --- | --- | --- | --- |
| C10-C40 | 0.44*** |  |  |  |  |  |
| pH | -0.15 | -0.21 |  |  |  |  |
| TOM | 0.68*** | 0.24* | -0.38** |  |  |  |
| Longitude | -0.42*** | -0.26* | -0.53*** | -0.07 |  |  |
| Latitude | 0.31** | 0.22* | -0.41*** | 0.02 | -0.03 |  |
| DNA concentration | 0.75*** | 0.14 | -0.34** | 0.74*** | -0.10 | 0.17 |

Significance level: ***** P < 0.001, **** P < 0.01, *** P < 0.05.

**Supplementary Table S4.** Distance-based redundancy analysis of individual single variable (marginal test) and the final model fitted

| Ecosystem | db-RDA test | Environmental variables | Proportion | F | Sig. |
| --- | --- | --- | --- | --- | --- |
| Sediment (in both seasons) | Marginal test | DNA concentration | 3.97 % | 2.68 | *** |
|  |  | C10-C40 | 3.98 % | 2.69 | *** |
|  |  | pH | 3.56 % | 2.40 | ** |
|  |  | Conductivity | 4.08 % | 2.77 | *** |
|  |  | TOM | 4.19 % | 2.84 | *** |
|  | Final model | Bray-Curtis distance ~ DNA concentration + C10-C40 + pH + Conductivity + TOM | 17.81 % | 2.64 | *** |
| Water (in both seasons) | Marginal test | Salinity | 3.36 % | 4.19 | ** |
|  |  | Conductivity | 3.00 % | 4.27 | ** |
|  |  | DNA concentration | 5.11 % | 3.18 | ** |
|  |  | pH | 5.04 % | 4.70 | *** |
|  | Final model | Bray-Curtis distance ~ Salinity + DNA concentration + pH | 21.28 % | 5.79 | *** |
| Water (only in summer) | Marginal test | Salinity | 7.46 % | 4.19 | ** |
|  |  | Conductivity | 7.59 % | 4.27 | *** |
|  |  | DNA concentration | 5.78 % | 3.18 | *** |
|  |  | pH | 10.01 % | 5.79 | *** |
|  |  | TOC | 6.96 % | 3.89 | ** |
|  | Final model | Bray-Curtis distance ~ Salinity + DNA concentration + pH | 25.42 % | 4.18 | *** |

Here the water samples included both coastal water and open sea water. As electrical conductivity and salinity were highly collinear in water samples, one of them was removed from the final model. Only the results of significant variables were reported. Proportion the proportion of variance explained by the single variable or the final model. Significance level (Sig.): ***** P < 0.001, **** P < 0.01, *** P < 0.05. *Proportion* represents the percentage of variance explained by the single variable or the final model.

**Supplementary Table S5.** The expected value of Moran's autocorrelation coefficient (Moran’s I) for relative abundance of archaeal major classes (relative abundance > 0.5%). The significant values (P < 0.05) were indicated in bold.

|  | Summer | | | Winter | | |
| --- | --- | --- | --- | --- | --- | --- |
| Ecosystem | S | OS | W | S | OS | W |
| c__Methanobacteria | -0.08 | -0.46 | -0.06 | 0.08 | -0.61 | -0.25 |
| c__Methanomicrobia | -0.13 | -0.30 | **0.34*** | -0.11 | -0.67 | **0.49***** |
| c__Thermococci | -0.01 | -0.67 | 0.07 | -0.33 | -0.63 | 0.02 |
| c__Methanococci | 0.04 | -0.67 | -0.02 | -0.06 | -0.48 | -0.02 |
| c__Thermoplasmata | -0.07 | -0.67 | -0.15 | -0.14 | -0.67 | -0.13 |
| c__Thermoprotei | -0.21 | -0.72 | 0.19 | -0.01 | -0.67 | -0.14 |
| c__Thaumarchaeota | -0.02 | -0.67 | -0.01 | 0.17 | -0.29 | -0.05 |
| c__Korarchaeota | -0.09 | -0.30 | -0.03 | -0.09 | NaN | -0.09 |
| c__Halobacteria | 0.24 | -0.52 | -0.45 | 0.00 | -0.64 | -0.37 |

Values close to -1 represent complete dispersal and close to 1 represent perfect spatial autocorrelation. The new archaeal phyla *Thaumarchaeota* and *Korarchaeota* are indicated as classes within the phylum *Crenarchaeota* in this study according to the Greengenes taxonomy reference database used. Significance level: ***** BH adjusted P < 0.001, *** BH adjusted P < 0.05. Abbreviations: *S* littoral sediment; *W* coastal water; *OS* open sea water.

**Supplementary Table S6.** Summary of hypothesis testing (Bray-Curtis distance-based discriminant analysis db-DA, PERMANOVA and Betadisper)

| *a priori* group | Data | Varm | *m* | Tot | Percent | Proportion of trace means | | db-DA perm. P | PERMANOVA perm. P | Betadisper perm. P |
| --- | --- | --- | --- | --- | --- | --- | --- | --- | --- | --- |
|  |  |  |  |  |  | LD1 | LD2 |  |  |  |
| Ecosystem | all | 61.93 | 8 | 67.09 | 83.06 | 0.95 | 0.05 | *** | *** | *** |
| EcoSeason | all | 66.89 | 10 | 67.09 | 57.38 | 0.73 | 0.20 | *** | *** | *** |
| Season | S | 61.79 | 10 | 32.72 | 72.94 | 1.00 | 0.00 | * | ** | 0.073 |
|  | OS | 72.25 | 2 | 4.32 | 82.35 | 1.00 | 0.00 | * | ** | 0.640 |
|  | W | 55.07 | 3 | 21.38 | 82.72 | 1.00 | 0.00 | *** | *** | *** |
| OilSeason | S | 61.78 | 10 | 32.72 | 64.71 | 0.67 | 0.26 | *** | *** | * |
|  | W | 55.07 | 3 | 21.38 | 74.07 | 0.93 | 0.64 | *** | *** | *** |
| Oil detection | S | 55.57 | 8 | 32.72 | 85.88 | 1.00 | 0.00 | *** | *** | 0.156 |
|  | W | 30.60 | 1 | 21.38 | 86.42 | ˗ | ˗ | 0.086 | 0.101 | 0.977 |

Significance level: ***** P < 0.001, **** P < 0.01, *** P < 0.05. Abbreviations: *EcoSeason* a combined factor of ecosystem and season; *OilSeason* a combined factor of oil detection and season; *S* littoral sediment; *W* coastal water; *OS* open sea water; *m* the number of axes analyzed by discriminant analysis; *Varm* the variance of the *m* axes that were investigated; *Tot* the total variance (sum of all eigenvalues of PCoA); *Percent* Percentage of correct classifications; *Proportion of trace means* the percentage of the between-group variance between *a priori* groups can be explained using the first (LD1) or second (LD2) axis; *perm. P* P-values calculated based on permutation.

**Supplementary Table S7.** Taxonomy of the core OTUs (nodes) that constructed the oil-contaminated and the clean sediment pMENs

| OTU ID | Contaminated pMEN | Clean pMEN | Taxonomy | | | | | |
| --- | --- | --- | --- | --- | --- | --- | --- | --- |
|  | Module No. | Module No. | phylum | class | Order | Family | Genus | Species |
| **001** | 2 | 1 | p__Euryarchaeota | c__Methanobacteria | o__Methanobacteriales | f__Methanobacteriaceae | g__Methanothermobacter | s__tenebrarum |
| **002** | 1 | 1 | p__Crenarchaeota | c__Thaumarchaeota | o__D-F10 | o__D-F10_unclassified | o__D-F10_unclassified | o__D-F10_unclassified |
| **003** | 3 | 3 | p__Euryarchaeota | c__Halobacteria | o__Halobacteriales | f__Halobacteriaceae | g__Halobacteriaceae | s__XD46 |
| **005** | 3 | 1 | p__Euryarchaeota | c__Halobacteria | o__Halobacteriales | f__Halobacteriaceae | g__Halobacteriaceae | s__XDS2 |
| **006** | 1 | 3 | p__Euryarchaeota | c__Methanococci | o__Methanococcales | f__Methanocaldococcaceae | g__Methanocaldococcus | g__Methanocaldococcus_unclassified |
| **007** | 1 | 1 | p__Euryarchaeota | c__Halobacteria | o__Halobacteriales | f__MSP41 | f__MSP41_unclassified | f__MSP41_unclassified |
| **008** | 3 | 2 | p__Euryarchaeota | c__Methanobacteria | o__Methanobacteriales | f__Methanobacteriaceae | g__Methanothermobacter | s__tenebrarum |
| **010** | 3 | 2 | p__Euryarchaeota | c__Thermoplasmata | o__Thermoplasmatales | f__Picrophilaceae | g__Picrophilus | s__torridus |
| **012** | 3 | 1 | p__Euryarchaeota | c__Methanomicrobia | o__Methanosarcinales | f__Methanosarcinaceae | g__Methanohalobium | s__evestigatum |
| **013** | 3 | 1 | p__Crenarchaeota | c__Thermoprotei | o__Thermoproteales | f__Thermoproteaceae | g__Vulcanisaeta | s__distributa |
| **014** | 3 | 2 | p__Crenarchaeota | c__Thermoprotei | o__Thermoproteales | f__Thermoproteaceae | g__Acidilobus | s__saccharovorans |
| **015** | 3 | 2 | p__Crenarchaeota | c__Thermoprotei | o__Sulfolobales | f__Sulfolobaceae | g__Sulfolobus | s__tengchongensis |
| **016** | 2 | 1 | p__Euryarchaeota | c__Halobacteria | o__Halobacteriales | f__Halobacteriaceae | g__haloarchaeon | s__98NT4 |
| **017** | 2 | 3 | p__Euryarchaeota | c__Methanomicrobia | o__Methanomicrobiales | f__Methanomicrobiaceae | g__Methanofollis | s__liminatans |
| **019** | 1 | 3 | p__Euryarchaeota | c__Halobacteria | o__Halobacteriales | f__MSP41 | f__MSP41_unclassified | f__MSP41_unclassified |
| **021** | 1 | 1 | p__Euryarchaeota | c__Methanobacteria | o__Methanobacteriales | f__Methanobacteriaceae | g__Methanosphaera | g__Methanosphaera_unclassified |
| **022** | 2 | 3 | p__Euryarchaeota | c__Halobacteria | o__Halobacteriales | f__Halobacteriaceae | g__XKL75 | g__XKL75_unclassified |
| **023** | 1 | 3 | p__Euryarchaeota | c__Methanococci | o__Methanococcales | f__Methanocaldococcaceae | g__Methanocaldococcus | g__Methanocaldococcus_unclassified |
| **024** | 1 | 1 | p__Euryarchaeota | c__Halobacteria | o__Halobacteriales | f__MSP41 | f__MSP41_unclassified | f__MSP41_unclassified |
| **026** | 2 | 2 | p__Euryarchaeota | c__Thermococci | o__Thermococcales | f__Thermococcaceae | g__Thermococcus | s__litoralis |
| **028** | 1 | 3 | p__Euryarchaeota | c__Halobacteria | o__Halobacteriales | f__Halobacteriaceae | g__Halobacteriaceae | s__XD48 |
| **029** | 3 | 1 | p__Euryarchaeota | c__Halobacteria | o__Halobacteriales | f__Halobacteriaceae | g__haloarchaeon | s__98NT4 |
| **032** | 2 | 2 | p__Euryarchaeota | c__Halobacteria | o__Halobacteriales | f__Halobacteriaceae | g__Halobiforma | s__lacisalsi |
| **033** | 2 | 2 | p__Euryarchaeota | c__Thermoplasmata | o__Thermoplasmatales | f__A10 | f__A10_unclassified | f__A10_unclassified |
| **034** | 3 | 2 | p__Euryarchaeota | c__Halobacteria | o__Halobacteriales | f__Halobacteriaceae | g__Halococcus | s__hamelinensis |
| **035** | 2 | 2 | p__Euryarchaeota | c__Thermococci | o__Thermococcales | f__Thermococcaceae | g__Thermococcus | s__litoralis |
| **036** | 3 | 2 | p__Euryarchaeota | c__Thermococci | o__Thermococcales | f__Thermococcaceae | g__Thermococcus | s__litoralis |
| **037** | 3 | 3 | p__Euryarchaeota | c__Halobacteria | o__Halobacteriales | f__Halobacteriaceae | g__Halopiger | s__xanaduensis |
| **039** | 1 | 1 | p__Euryarchaeota | c__Methanomicrobia | o__Methanomicrobiales | f__Methanocorpusculaceae | g__Methanocorpusculum | g__Methanocorpusculum_unclassified |
| **042** | 3 | 2 | p__Euryarchaeota | c__Halobacteria | o__Halobacteriales | f__Halobacteriaceae | g__Halovivax | s__ruber |
| **047** | 3 | 3 | p__Euryarchaeota | c__Thermoplasmata | o__E2 | f__[Methanomassiliicoccaceae] | g__Methanomassiliicoccus | g__Methanomassiliicoccus_unclassified |
| **048** | 2 | 1 | p__Euryarchaeota | c__Thermococci | o__Thermococcales | f__Thermococcaceae | g__Thermococcus | s__litoralis |
| **052** | 3 | 3 | p__Euryarchaeota | c__Halobacteria | o__Halobacteriales | f__Halobacteriaceae | g__Halovivax | s__asiaticus |
| **053** | 3 | 2 | p__Euryarchaeota | c__Halobacteria | o__Halobacteriales | f__Halobacteriaceae | g__Halobacteriaceae | s__YC21 |
| **054** | 2 | 2 | p__Euryarchaeota | c__Methanomicrobia | o__Methanomicrobiales | f__Methanomicrobiaceae | g__Methanofollis | s__liminatans |
| **058** | 3 | 1 | p__Euryarchaeota | c__Thermococci | o__Thermococcales | f__Thermococcaceae | g__Thermococcus | s__litoralis |
| **059** | 3 | 2 | p__Euryarchaeota | c__Halobacteria | o__Halobacteriales | f__Halobacteriaceae | g__Halomicrobium | g__Halomicrobium_unclassified |
| **060** | 1 | 2 | p__Euryarchaeota | c__Thermococci | o__Thermococcales | f__Thermococcaceae | g__Thermococcus | s__gammatolerans |
| **062** | 1 | 3 | p__Euryarchaeota | c__Halobacteria | o__Halobacteriales | f__Halobacteriaceae | g__Halovivax | g__Halovivax_unclassified |
| **063** | 2 | 2 | p__Crenarchaeota | c__MHVG | c__MHVG_unclassified | c__MHVG_unclassified | c__MHVG_unclassified | c__MHVG_unclassified |
| **064** | 2 | 3 | p__Euryarchaeota | c__Halobacteria | o__Halobacteriales | f__Halobacteriaceae | g__Natrialba | s__hulunbeirensis |
| **072** | 3 | 3 | p__Euryarchaeota | c__Thermoplasmata | o__E2 | f__[Methanomassiliicoccaceae] | g__Methanomassiliicoccus | g__Methanomassiliicoccus_unclassified |
| **073** | 3 | 3 | p__Euryarchaeota | c__Halobacteria | o__Halobacteriales | f__Halobacteriaceae | g__Halobacteriaceae | s__GC6 |
| **079** | 2 | 1 | p__Euryarchaeota | c__Halobacteria | o__Halobacteriales | f__Halobacteriaceae | g__Halobacteriaceae | s__XDS2 |
| **090** | 3 | 2 | p__Euryarchaeota | c__Halobacteria | o__Halobacteriales | f__Halobacteriaceae | g__halophilic | s__MK13-1 |
| **091** | 2 | 1 | p__Euryarchaeota | c__Halobacteria | o__Halobacteriales | f__Halobacteriaceae | g__Halovivax | g__Halovivax_unclassified |
| **093** | 1 | 3 | k__Archaea_unclassified | k__Archaea_unclassified | k__Archaea_unclassified | k__Archaea_unclassified | k__Archaea_unclassified | k__Archaea_unclassified |
| **096** | 2 | 1 | p__Euryarchaeota | c__Halobacteria | o__Halobacteriales | f__Halobacteriaceae | g__Halopiger | s__xanaduensis |
| **0101** | 1 | 2 | p__Euryarchaeota | c__Thermococci | o__Thermococcales | f__Thermococcaceae | g__Thermococcus | s__litoralis |
| **0117** | 2 | 2 | p__Euryarchaeota | c__Halobacteria | o__Halobacteriales | f__Halobacteriaceae | g__Halobacteriaceae | s__GC6 |
| 04 |  | 2 | k__Archaea_unclassified | k__Archaea_unclassified | k__Archaea_unclassified | k__Archaea_unclassified | k__Archaea_unclassified | k__Archaea_unclassified |
| 030 |  | 3 | p__Euryarchaeota | c__Methanomicrobia | o__Methanosarcinales | f__Methermicoccaceae | f__Methermicoccaceae_unclassified | f__Methermicoccaceae_unclassified |
| 040 |  | 3 | p__Euryarchaeota | c__Thermococci | o__Thermococcales | f__Thermococcaceae | g__Thermococcus | s__gammatolerans |
| 044 |  | 3 | p__Euryarchaeota | c__Halobacteria | o__Halobacteriales | f__Halobacteriaceae | g__Halovivax | g__Halovivax_unclassified |
| 045 |  | 1 | p__Euryarchaeota | c__Thermoplasmata | o__E2 | f__[Methanomassiliicoccaceae] | g__Methanomassiliicoccus | g__Methanomassiliicoccus_unclassified |
| 046 |  | 1 | p__Euryarchaeota | c__Halobacteria | o__Halobacteriales | f__Halobacteriaceae | g__Halopiger | s__xanaduensis |
| 049 |  | 1 | p__Euryarchaeota | c__Halobacteria | o__Halobacteriales | f__Halobacteriaceae | g__haloarchaeon | s__98NT4 |
| 051 |  | 3 | p__Euryarchaeota | c__Halobacteria | o__Halobacteriales | f__Halobacteriaceae | g__Halostagnicola | s__larsenii |
| 075 |  | 3 | p__Euryarchaeota | c__Thermococci | o__Thermococcales | f__Thermococcaceae | g__Thermococcus | s__litoralis |
| 077 |  | 1 | p__Euryarchaeota | c__Halobacteria | o__Halobacteriales | f__Halobacteriaceae | g__Halovivax | s__asiaticus |
| 080 |  | 1 | p__Euryarchaeota | c__Halobacteria | o__Halobacteriales | f__MSP41 | f__MSP41_unclassified | f__MSP41_unclassified |
| 081 |  | 1 | p__Euryarchaeota | c__Halobacteria | o__Halobacteriales | f__Halobacteriaceae | g__haloarchaeon | s__98NT4 |
| 086 |  | 1 | p__Euryarchaeota | c__Thermococci | o__Thermococcales | f__Thermococcaceae | g__Thermococcus | s__litoralis |
| 092 |  | 3 | p__Euryarchaeota | c__Thermococci | o__Thermococcales | f__Thermococcaceae | g__Thermococcus | s__gammatolerans |
| 097 |  | 3 | p__Euryarchaeota | c__Halobacteria | o__Halobacteriales | f__Halobacteriaceae | g__Halobacteriaceae | s__GC6 |
| 099 |  | 2 | p__Crenarchaeota | c__Thermoprotei | o__Thermoproteales | f__Thermoproteaceae | g__Caldivirga | s__maquilingensis |
| 105 |  | 3 | p__Euryarchaeota | c__Thermoplasmata | o__E2 | f__[Methanomassiliicoccaceae] | g__Methanomassiliicoccus | g__Methanomassiliicoccus_unclassified |
| 106 |  | 1 | p__Crenarchaeota | c__Thermoprotei | o__Desulfurococcales | f__Desulfurococcaceae | g__Staphylothermus | s__hellenicus |
| 122 |  | 1 | p__Euryarchaeota | c__Halobacteria | o__Halobacteriales | f__Halobacteriaceae | g__Halorubrum | s__alkaliphilum |
| 126 |  | 2 | p__Euryarchaeota | c__Thermoplasmata | o__E2 | f__[Methanomassiliicoccaceae] | g__Methanomassiliicoccus | g__Methanomassiliicoccus_unclassified |
| 132 |  | 1 | p__Euryarchaeota | c__Halobacteria | o__Halobacteriales | f__Halobacteriaceae | g__Halobellus | s__clavatus |
| 141 |  | 3 | p__Euryarchaeota | c__Halobacteria | o__Halobacteriales | f__Halobacteriaceae | g__halophilic | s__MK13-1 |
| 146 |  | 2 | p__Euryarchaeota | c__Thermococci | o__Thermococcales | f__Thermococcaceae | g__Thermococcus | s__gammatolerans |
| 147 |  | 2 | p__Euryarchaeota | c__Thermoplasmata | o__Thermoplasmatales | f__Picrophilaceae | g__Ferroplasma | s__cupricumulans |
| 151 |  | 1 | p__Euryarchaeota | c__Halobacteria | o__Halobacteriales | f__Halobacteriaceae | g__Halopiger | s__xanaduensis |
| 158 |  | 1 | p__Crenarchaeota | c__Thermoprotei | o__Desulfurococcales | f__Desulfurococcaceae | g__Thermogladius | s__cellulolyticus |
| 174 |  | 1 | p__Euryarchaeota | c__Halobacteria | o__Halobacteriales | f__Halobacteriaceae | g__haloarchaeon | s__98NT4 |
| 179 |  | 1 | p__Euryarchaeota | c__Thermococci | o__Thermococcales | f__Thermococcaceae | g__Thermococcus | s__litoralis |
| 185 |  | 3 | p__Euryarchaeota | c__Halobacteria | o__Halobacteriales | f__Halobacteriaceae | g__halophilic | s__MK13-1 |
| 186 |  | 1 | p__Euryarchaeota | c__Halobacteria | o__Halobacteriales | f__Halobacteriaceae | g__Halovivax | g__Halovivax_unclassified |
| 257 |  | 2 | p__Euryarchaeota | c__Halobacteria | o__Halobacteriales | f__Halobacteriaceae | g__halophilic | s__MK206-1 |
| 277 |  | 1 | p__Euryarchaeota | c__Halobacteria | o__Halobacteriales | f__Halobacteriaceae | g__Halovivax | g__Halovivax_unclassified |
| 09 | 3 |  | p__Euryarchaeota | c__Halobacteria | o__Halobacteriales | f__Halobacteriaceae | g__Halobacteriaceae | s__XD46 |
| 020 | 3 |  | p__Euryarchaeota | c__Halobacteria | o__Halobacteriales | f__Halobacteriaceae | g__Halobacteriaceae | s__XD46 |
| 025 | 3 |  | p__Euryarchaeota | c__Halobacteria | o__Halobacteriales | f__Halobacteriaceae | g__Halobacteriaceae | s__XD46 |
| 027 | 3 |  | p__Euryarchaeota | c__Halobacteria | o__Halobacteriales | f__Halobacteriaceae | g__Halobacteriaceae | s__XD46 |
| 041 | 1 |  | p__Crenarchaeota | c__Thermoprotei | o__Thermoproteales | f__Thermoproteaceae | g__Vulcanisaeta | s__distributa |
| 043 | 3 |  | p__Euryarchaeota | c__Methanobacteria | o__Methanobacteriales | f__Methanobacteriaceae | g__Methanothermobacter | s__tenebrarum |
| 050 | 1 |  | p__Euryarchaeota | c__Halobacteria | o__Halobacteriales | f__MSP41 | f__MSP41_unclassified | f__MSP41_unclassified |
| 056 | 1 |  | p__Euryarchaeota | c__Halobacteria | o__Halobacteriales | f__Halobacteriaceae | g__Halobacteriaceae | s__R22 |
| 061 | 2 |  | p__Euryarchaeota | c__Halobacteria | o__Halobacteriales | f__Halobacteriaceae | g__Halobacteriaceae | s__GC6 |
| 065 | 2 |  | p__Euryarchaeota | c__Thermococci | o__Thermococcales | f__Thermococcaceae | g__Thermococcus | s__litoralis |
| 067 | 3 |  | p__Euryarchaeota | c__Halobacteria | o__Halobacteriales | f__Halobacteriaceae | g__Halobacteriaceae | s__GC9 |
| 068 | 3 |  | p__Euryarchaeota | c__Methanomicrobia | o__Methanomicrobiales | f__Methanomicrobiaceae | g__Methanofollis | s__aquaemaris |
| 070 | 1 |  | p__Euryarchaeota | p__Euryarchaeota_unclassified | p__Euryarchaeota_unclassified | p__Euryarchaeota_unclassified | p__Euryarchaeota_unclassified | p__Euryarchaeota_unclassified |
| 076 | 2 |  | p__Euryarchaeota | c__Halobacteria | o__Halobacteriales | f__Halobacteriaceae | g__209ZB06 | g__209ZB06_unclassified |
| 083 | 1 |  | p__Euryarchaeota | c__Halobacteria | o__Halobacteriales | f__Halobacteriaceae | g__Halobacteriales | s__YIM_93590 |
| 084 | 2 |  | p__Euryarchaeota | c__Halobacteria | o__Halobacteriales | f__Halobacteriaceae | g__Natrialba | s__hulunbeirensis |
| 085 | 2 |  | p__Euryarchaeota | c__Halobacteria | o__Halobacteriales | f__Halobacteriaceae | g__Halovivax | s__asiaticus |
| 089 | 3 |  | p__Euryarchaeota | c__Thermococci | o__Thermococcales | f__Thermococcaceae | g__Thermococcus | s__litoralis |
| 095 | 1 |  | p__Euryarchaeota | c__Halobacteria | o__Halobacteriales | f__Halobacteriaceae | g__haloarchaeon | s__98NT4 |
| 100 | 3 |  | p__Euryarchaeota | c__Thermococci | o__Thermococcales | f__Thermococcaceae | g__Thermococcus | s__litoralis |
| 102 | 1 |  | p__Crenarchaeota | c__MHVG | c__MHVG_unclassified | c__MHVG_unclassified | c__MHVG_unclassified | c__MHVG_unclassified |
| 104 | 1 |  | p__Euryarchaeota | c__Halobacteria | o__Halobacteriales | f__Halobacteriaceae | g__Halovivax | g__Halovivax_unclassified |
| 110 | 2 |  | p__Euryarchaeota | c__Thermococci | o__Thermococcales | f__Thermococcaceae | g__Thermococcus | s__zilligii |
| 116 | 2 |  | p__Euryarchaeota | c__Halobacteria | o__Halobacteriales | f__Halobacteriaceae | g__Halobacteriaceae | s__SL-2 |
| 121 | 2 |  | p__[Parvarchaeota] | c__[Micrarchaea] | o__[Micrarchaeles] | o__[Micrarchaeles]_unclassified | o__[Micrarchaeles]_unclassified | o__[Micrarchaeles]_unclassified |
| 125 | 3 |  | p__Euryarchaeota | c__Halobacteria | o__Halobacteriales | f__Halobacteriaceae | g__Halobacteriaceae | s__XD46 |
| 127 | 1 |  | p__Euryarchaeota | c__Halobacteria | o__Halobacteriales | f__Halobacteriaceae | g__haloarchaeon | s__98NT4 |
| 128 | 2 |  | p__Euryarchaeota | c__Halobacteria | o__Halobacteriales | f__Halobacteriaceae | g__Halovivax | s__ruber |
| 133 | 1 |  | p__Euryarchaeota | p__Euryarchaeota_unclassified | p__Euryarchaeota_unclassified | p__Euryarchaeota_unclassified | p__Euryarchaeota_unclassified | p__Euryarchaeota_unclassified |
| 135 | 2 |  | p__Euryarchaeota | c__Halobacteria | o__Halobacteriales | f__Halobacteriaceae | g__Natrialba | s__aegyptia |
| 138 | 1 |  | p__Euryarchaeota | c__Thermococci | o__Thermococcales | f__Thermococcaceae | g__Thermococcus | s__litoralis |
| 142 | 1 |  | p__Euryarchaeota | c__Thermococci | o__Thermococcales | f__Thermococcaceae | g__Thermococcus | s__litoralis |
| 143 | 2 |  | p__Euryarchaeota | c__Halobacteria | o__Halobacteriales | f__Halobacteriaceae | f__Halobacteriaceae_unclassified | f__Halobacteriaceae_unclassified |
| 144 | 1 |  | p__Euryarchaeota | c__Methanococci | o__Methanococcales | f__Methanocaldococcaceae | g__Methanocaldococcus | g__Methanocaldococcus_unclassified |
| 150 | 2 |  | p__Euryarchaeota | c__Halobacteria | o__Halobacteriales | f__Halobacteriaceae | g__Halovivax | s__ruber |
| 159 | 2 |  | p__Euryarchaeota | c__Halobacteria | o__Halobacteriales | f__Halobacteriaceae | g__Halobacteriaceae | s__GC6 |
| 163 | 1 |  | p__Euryarchaeota | c__Thermococci | o__Thermococcales | f__Thermococcaceae | g__Thermococcus | s__litoralis |
| 168 | 3 |  | p__Crenarchaeota | c__Thermoprotei | o__Thermoproteales | f__Thermoproteaceae | g__Vulcanisaeta | s__distributa |
| 176 | 2 |  | k__Archaea_unclassified | k__Archaea_unclassified | k__Archaea_unclassified | k__Archaea_unclassified | k__Archaea_unclassified | k__Archaea_unclassified |
| 181 | 1 |  | p__Euryarchaeota | c__Halobacteria | o__Halobacteriales | f__Halobacteriaceae | g__Halovivax | g__Halovivax_unclassified |
| 194 | 3 |  | p__Euryarchaeota | c__Halobacteria | o__Halobacteriales | f__Halobacteriaceae | g__Halopiger | s__xanaduensis |
| 233 | 2 |  | p__Euryarchaeota | c__Thermococci | o__Thermococcales | f__Thermococcaceae | g__Thermococcus | s__zilligii |
| 238 | 1 |  | p__Crenarchaeota | c__Korarchaeota | o__pJP78 | o__pJP78_unclassified | o__pJP78_unclassified | o__pJP78_unclassified |

The OTUs that were shared by both pMENs are presented in bold. The new archaeal phyla *Thaumarchaeota* and *Korarchaeota* are indicated as classes within the phylum *Crenarchaeota* in this study according to the Greengenes taxonomy reference database used.

**2. Supplementary Figures**


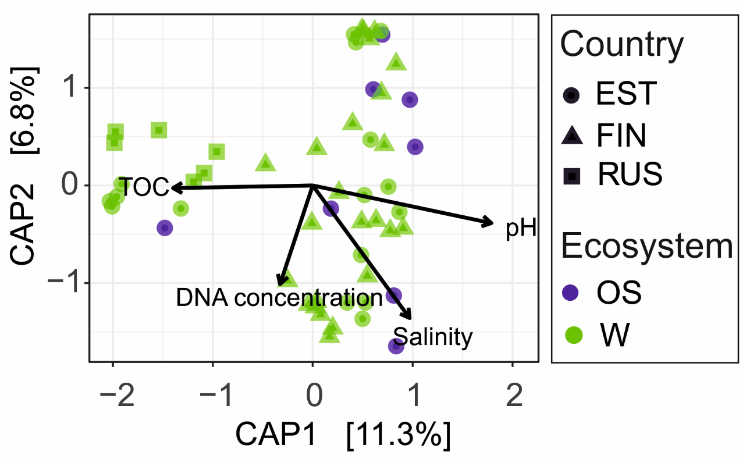


**Supplementary Figure S1.** Visualization of the relationships between the observed water archaeal communities and measured environmental variables in summer, based on a distance-based redundancy analysis (db-RDA, scaling=2). The marginal test of individual environmental variable on the variations of archaeal communities can refer to Supplementary Supplementary Table S6. Abbreviations: *EST* Estonia; *FIN* Finland; *RUS* Russia; *W* coastal water; *OS* open sea water; *BDL* Oil concentration below detection level (clean samples); *Detected* oil detected (oil-contaminated samples).


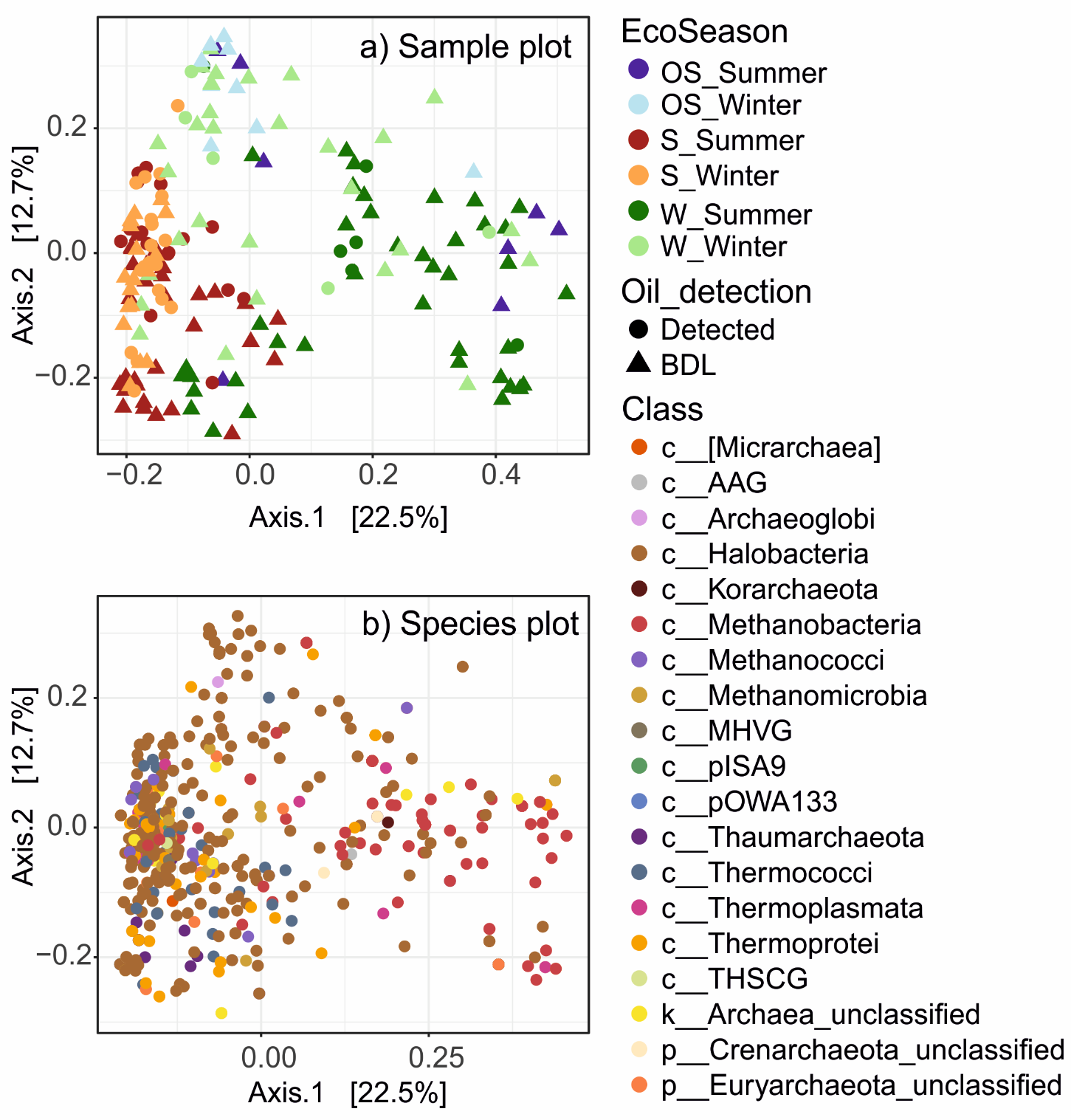


**Supplementary Figure S2.** Principal Coordinates Analysis (PCoA) based on weighted UniFrac distance, providing visualization on the distribution patterns of a) observed communities (samples) and b) OTUs (colored by classes). Abbreviations: *EcoSeason* a combined factor of ecosystem and season; *S* littoral sediment; *W* coastal water; *OS* open sea water; *BDL* Oil concentration below detection level (clean samples); *Detected* oil detected (oil-contaminated samples).


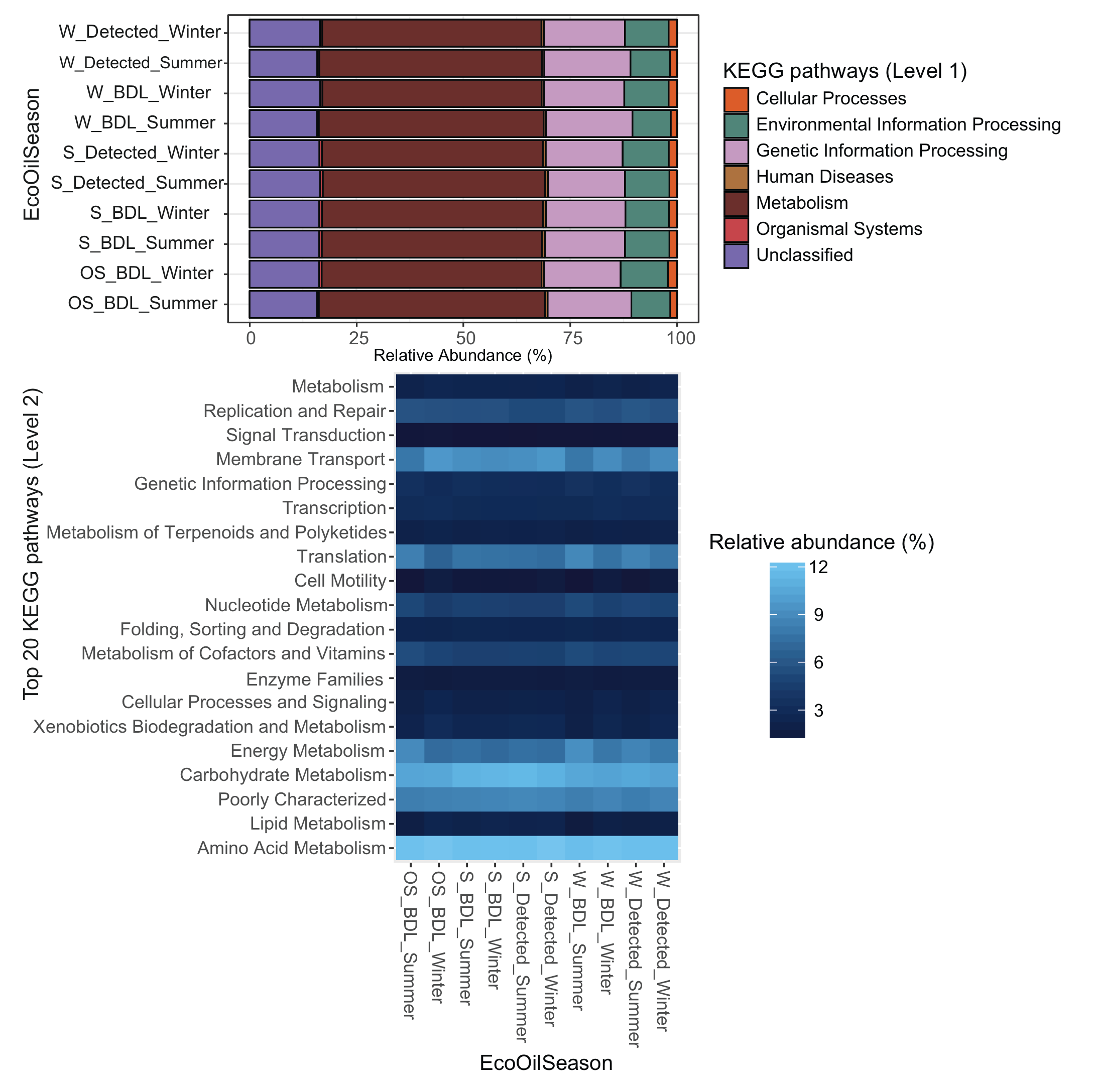


**Supplementary Figure S3.** Predicted KEGG pathways. Abbreviations: *EcoOilSeason* a combined factor of ecosystem, oil detection and season; *S* littoral sediment; *W* coastal water; *OS* open sea water; *BDL* Oil concentration below detection level (clean samples); *Detected* oil detected (oil-contaminated samples).
